# Selective Cannabinoid 2 Receptor Agonists as Potential Therapeutic Drugs for the Treatment of Endotoxin-Induced Uveitis

**DOI:** 10.3390/molecules24183338

**Published:** 2019-09-13

**Authors:** Richard Frederick Porter, Anna-Maria Szczesniak, James Thomas Toguri, Simon Gebremeskel, Brent Johnston, Christian Lehmann, Jürgen Fingerle, Benno Rothenhäusler, Camille Perret, Mark Rogers-Evans, Atsushi Kimbara, Matthias Nettekoven, Wolfgang Guba, Uwe Grether, Christoph Ullmer, Melanie E. M. Kelly

**Affiliations:** 1 Department of Pharmacology, Dalhousie University, Halifax, NS B3H 4R2, Canada; richard.porter@dal.ca (R.F.P.); Anna.Maria.Szczesniak@dal.ca (A.-M.S.); ttoguri@dal.ca (J.T.T.); chlehmann@dal.ca (C.L.); 2 Department of Microbiology and Immunology, Dalhousie University, Halifax, NS B3H 4R2, Canada; rassimon@gmail.com (S.G.); brent.johnston@dal.ca (B.J.); 3 Department of Pediatrics, Dalhousie University, Halifax, NS B3H 4R2, Canada; 4 Department of Anesthesia, Pain Management and Perioperative Care, Dalhousie University, Halifax, NS B3H 4R2, Canada; 5 Roche Innovation Center Basel, F. Hoffmann-La Roche Ltd., 4070 Basel, Switzerland; juergen.fingerle@roche.com (J.F.); benno.rothenhaeusler@roche.com (B.R.); camille.perret@roche.com (C.P.); mark.rogers-evans@roche.com (M.R.-E.); atsushi.kimbara@roche.com (A.K.); matthias.nettekoven@roche.com (M.N.); wolfgang.guba@roche.com (W.G.); uwe.grether@roche.com (U.G.); christoph.ullmer@roche.com (C.U.); 6 Department of Ophthalmology and Visual Sciences, Dalhousie University, Halifax, NS B3H 2Y9, Canada

**Keywords:** cannabinoid 2 receptor, synthetic cannabinoids, selective cannabinoid ligands, structure-activity relationship, uveitis, anti-inflammatory

## Abstract

(1) Background: The cannabinoid 2 receptor (CB_2_R) is a promising anti-inflammatory drug target and development of selective CB_2_R ligands may be useful for treating sight-threatening ocular inflammation. (2) Methods: This study examined the pharmacology of three novel chemically-diverse selective CB_2_R ligands: CB_2_R agonists, RO6871304, and RO6871085, as well as a CB_2_R inverse agonist, RO6851228. In silico molecular modelling and *in vitro* cell-based receptor assays were used to verify CB_2_R interactions, binding, cell signaling (ß-arrestin and cAMP) and early absorption, distribution, metabolism, excretion, and toxicology (ADMET) profiling of these receptor ligands. All ligands were evaluated for their efficacy to modulate leukocyte-neutrophil activity, in comparison to the reported CB_2_R ligand, HU910, using an *in vivo* mouse model of endotoxin-induced uveitis (EIU) in wild-type (WT) and CB_2_R^-/-^ mice. The actions of RO6871304 on neutrophil migration and adhesion were examined *in vitro* using isolated neutrophils from WT and CB_2_R^-/-^ mice, and *in vivo* in WT mice with EIU using adoptive transfer of WT and CB_2_R^-/-^ neutrophils, respectively. (3) Results: Molecular docking studies indicated that RO6871304 and RO6871085 bind to the orthosteric site of CB_2_R. Binding studies and cell signaling assays for RO6871304 and RO6871085 confirmed high-affinity binding to CB_2_R and selectivity for CB_2_R > CB_1_R, with both ligands acting as full agonists in cAMP and ß-arrestin assays (EC_50_s in low nM range). When tested in EIU, topical application of RO6871304 and RO6871085 decreased leukocyte-endothelial adhesion and this effect was antagonized by the inverse agonist, RO6851228. The CB_2_R agonist, RO6871304, decreased *in vitro* neutrophil migration of WT neutrophils but not neutrophils from CB_2_R^-/-^, and attenuated adhesion of adoptively-transferred leukocytes in EIU. (4) Conclusions: These unique ligands are potent and selective for CB_2_R and have good immunomodulating actions in the eye. RO6871304 and RO6871085, as well as HU910, decreased leukocyte adhesion in EIU through inhibition of resident ocular immune cells. The data generated with these three structurally-diverse and highly-selective CB_2_R agonists support selective targeting of CB_2_R for treating ocular inflammatory diseases.

## 1. Introduction

Uveitis is a heterogeneous group of ocular inflammatory diseases and is estimated to account for 10% of blindness in developed countries [1]. Uveitis involves the iris, ciliary body, and choroid and can be classified as anterior, intermediate, posterior, or panuveitis, depending on which area of the eye is inflamed. Although autoimmunity or infection are the main causes of uveitis, in many patients the etiology of uveitis is idiopathic [2]. Endotoxin-induced uveitis (EIU) in mice mimics human panuveitis and is induced by lipopolysaccharide (LPS), a component of a gram-negative bacterial cell wall, which activates inflammatory Toll-like receptor 4 (TLR4) signaling [3,4]. The mainstay drugs used to treat uveitis include topical and systemic steroids; however, steroid use has many adverse systemic and ocular effects [5]. Identification of new drug targets is necessary to treat ocular inflammation [6].

One potential target to modulate inflammation is the endocannabinoid system [7,8,9]. The endocannabinoid system is an endogenous lipid-signaling system comprised of two G protein-coupled receptors, cannabinoid 1 receptor (CB_1_R) and cannabinoid 2 receptor (CB_2_R), and enzymes responsible for endocannabinoid synthesis and degradation [10,11,12]. The two most well-studied endocannabinoids are *N*-arachidonoyl ethanolamine (AEA) and 2-arachidonoyl glycerol (2-AG), both of which act as non-selective agonists at CB_1_R and CB_2_R. Activation of either CB_1_R or CB_2_R results in downstream coupling to G proteins, including G_i/o_, with resultant modulation of intracellular signal-transduction pathways, such as inhibition of adenylyl cyclase and stimulation of mitogen-activated protein kinases. CB_1_R is highly expressed in the central nervous system, and activation of this receptor regulates the presynaptic release of neurotransmitters, resulting in alterations in pain perception, motor behavior, learning, and memory. CB_2_R is almost exclusively expressed on immune cells and non-neural cells [13,14], and drugs acting at this receptor lack psychotropic side-effects associated with cannabinoids that act at CB_1_R [15].

The presence of CB_2_R on both innate and adaptive immune cells suggests that this receptor may be a promising target for modulation of the immune response [16,17]. CB_2_R mRNA expression is highly induced during inflammatory conditions [18,19], and activation of CB_2_R has been reported to have anti-inflammatory effects in experimental models of Parkinson’s disease, Huntington’s disease, multiple sclerosis, stroke, atherosclerosis, and other inflammatory disorders [7,20,21]. Work from our group, using models of experimental EIU and proliferative vitreoretinopathy, indicates increased CB_2_R expression and activity in the eye during ocular inflammation; antagonist/inverse agonists produce enhanced LPS-induced inflammation [22,23]. Additionally, CB_2_R receptor activation using exogenous synthetic cannabinoids reduces leukocyte adhesion in the iris microvasculature, decreases levels of ocular pro-inflammatory mediators, and reduces disease pathology [22,23,24]. 

The pharmacology of endocannabinoids and phytocannabinoids, such as Δ^9^-tetrahydrocannabinol, cannabidiol, and their derivatives is complex given that many of these ligands are non-selective for cannabinoid receptors and may also mediate some of their actions through non-cannabinoid receptors [25,26,27]. In addition, most synthetic cannabinoids that have been developed, including WIN55,212-2, CP55,940, HU308, JWH015, and SR141716a (rimonabant) exhibit non-CB_2_R- and CB_1_R-mediated activity [28]. For the purposes of target validation and drug development it is necessary to have synthetic agonists that are highly selective and have well-defined receptor signaling pathways with suitable “drug-like” properties for the intended use. A high-throughput screen was used to identify potent and selective CB_2_R agonists. Two hit clusters, the triazolopyrimidines and 2,4,5-trisubstituted pyridines, were elaborated via iterative design, synthesis, and optimization cycles into attractive lead series [29,30,31,32]. This hit to lead work was guided by assessment of potency at CB_2_R (cAMP assay) and selectivity for CB_2_R versus CB_1_R (cAMP assay) [33]. During subsequent lead optimization high potency for CB_2_R was combined with favorable absorption, distribution, metabolism, excretion, and toxicology (ADMET) properties culminating in the discovery of triazolopyrimidine RO6871304 [33] and 2,4,5-trisubstituted pyridines RO6871085 [32] and RO6851228 [32]. For the triazolopyrimidine series, a detailed structure–activity relationship (SAR), starting from a high-throughput screening hit toward CB_2_R agonists with enhanced potency and physicochemical properties, has been outlined by Nettekoven et al. (2016) [33].

The present study extends these results by reporting both the detailed ADMET profile (only partially reported in Nettekoven et al. (2016) [33] and in Alami et al. (2018) [32]) of RO6871304 and RO6871085, synthetic CB_2_R agonists that originate from two chemically-diverse series, and RO6851228, a structurally-related (to RO6871085) novel CB_2_R inverse agonist, as well as that of HU910 (only partially reported previously in Soethoudt et al. (2017) [28]), together with structurally-related HU308 [34], and addresses the pharmacology of these highly-potent and -selective CB_2_ ligands. Both *in vitro* and *in vivo* pharmacodynamic studies were undertaken in order to determine the suitability of these novel ligands as tool compounds for probing CB_2_R function and to evaluate whether targeting CB_2_R represents a rational approach for therapeutic management of ocular inflammatory disease, and with the long term goal of developing a CB_2_R agonist to address this unmet medical need.

## 2. Results

### 2.1. Chemical Structure 

The CB_2_R ligands used in this study originate from three structurally-diverse chemotypes (Table 1). RO6871304 is a representative of the triazolopyrimidine class and is a CB_2_R agonist [33]. RO6871085 [30] as well as RO6851228 [31] are 2,4,5-trisubstituted pyridines. While RO6871085 is a CB_2_R agonist, structurally-related RO6851228 exhibits inverse agonism at both mouse and human CB_2_R [32]. RO6851228 carries, at position 4, an oxetane residue that is slightly larger than the trifluorethoxy substituent of agonist RO6871085. Interestingly, this subtle structural change of the residue in position 4 seems to be responsible for the switch from agonism toward inverse agonism. Such effects have also been observed for further molecules from the 2,4,5-trisubstituted pyridine series with mouse CB_2_R being more susceptible to the agonism inverse agonism switch than human CB_2_R, suggesting that the agonist binding cavity of mouse CB_2_R might be smaller than that of human CB_2_R (unpublished results). The third chemotype is represented by HU910, a previously-published CB_2_R agonist, whose structure is similar to HU308 (also shown for comparison) but with a bicyclic camphor moiety replacing the pinene moiety [20].

Despite their structural diversity, all four ligands bind to the same orthosteric binding site of CB_2_R, as they all displace tritiated CP55,940 in a radioligand-binding assay. This is illustrated by overlaying the docking poses of RO6871304, RO6871085, and HU910 within the binding cavity of the inactive-state CB_2_R X-ray structure [35] (Supplemental Figure S1). The graph demonstrates that key ligand pharmacophore features for the different compound classes overlay and fit well into the binding cavity. Additionally, the potent inverse agonist, RO6851228, efficiently fills the binding pocket when docked into the inactive-state CB_2_R X-ray structure. These docking poses are supported by detailed SAR information on several hundred triazolopyrimidine and 2,4,5-trisubstituted pyridine ligands, which helped to identify the preferred orientation of the ligands (unpublished data). For example, replacing the position 5 *tert*-butyl residue of triazolopyrimidine RO6871304 by a difluorobenzyl moiety converts this ligand into a potent and selective CB_2_R inverse agonist. Elongation of the triazolopyrimidines at the tetrazole cyclopropyl substituent or the pyridines at the amide residue by a polyethylene glycol chain carrying a terminal reporter element, leads to high-affinity CB_2_R probes, thereby further narrowing down the options for different orientations of the ligands within the binding pocket. Irrespective of their functional activity, all ligands were docked into the inactive-state CB_2_R X-ray structure because this structure resembles more the X-ray structure of the active-state than of the inactive-state CB_1_R [36], making it very difficult to assume how the binding cavity of the active-state CB_2_R receptor will look like; generally GPCR active-state binding pockets are smaller than inactive-state binding pockets (approximately 50% for CB_1_R).

### 2.2. In Vitro Pharmacology of CB_2_R Ligands

The CB_2_R ligands were thoroughly evaluated in human and mouse CB_2_R-binding and functional *in vitro* pharmacology assays (partially published for HU308 and HU910 in [28], for RO6871304 in [29] and for RO6871085 as well as RO6851228 in [32]), starting with assessing the binding affinity on human and mouse CB_2_R as well as on human CB_1_R. A comparison of all data for these ligands is shown in Table 2. All molecules have a high binding affinity for the human CB_2_R. Triazolopyrimidine, RO6871304, was found to be the most potent at CB_2_R and exhibits a K_i_ of 17 nM. RO6871085 binding selectivity for human CB_2_R against the human CB_1_R was at least 44-fold. RO6871304 at concentrations up to 10 μM did not interact with CB_1_R and; therefore, had the most preferential binding selectivity (hCB_1_R K_i_/hCB_2_R K_i_ >588). Importantly, all molecules potently bind the mouse CB_2_R, although subtle species differences can be observed. While there was a decrease in binding affinity to mouse CB_2_R compared to human CB_2_R for HU308 (hCB_2_R K_i_/mouse CB_2_R K_i_ 0.04), HU910 (hCB_2_R K_i_/mouse CB_2_R K_i_ 0.2), and RO6871304 (hCB_2_R K_i_/mouse CB_2_ K_i_ 0.5), the 2,4,5-trisubstituted pyridines RO6871085 and RO6851228 were more potent in the mouse compared to the human binding assay (hCB_2_R K_i_/mouse CB_2_R K_i_ 13 and 45, respectively).

The functional activity and efficacy of ligands was assessed for human CB_2_R, mouse CB_2_R, and human CB_1_R using cAMP [37] and β-arrestin assays. In the cAMP assay, RO6871304 and RO6871085, like HU308 and HU910, were full agonists and possess low nanomolar EC_50_ values for the human CB_2_R. In contrast, RO6851228, a structurally-close analogue of RO6871085, is an inverse agonist with an EC_50_ value of 26 nM. Functional selectivities against CB_1_R were even higher than binding selectivities, reaching hCB_1_R cAMP EC_50_/hCB_2_R cAMP EC_50_ ratios > 10,000 in case of triazolopyrimidine, RO6871304. With respect to functionality no species differences existed between the agonists. Additionally, with the exception of RO6851228, all molecules were full agonists at the mouse CB_2_R. Human and mouse EC_50_ values were comparable; RO6871304 was the most potent molecule with a mouse CB_2_R cAMP EC_50_ value of 500 pM. RO6851228 was a slightly more potent inverse agonist for the mouse compared to the human CB_2_R, as indicated by its hCB_2_R cAMP EC_50_/mouse CB_2_R cAMP EC_50_ ratio of 7.

The ability of these CB_2_R ligands to recruit β-arrestin, which ultimately leads to the steric inhibition of G protein coupling and termination of signaling independent of G protein classes, was determined. RO6871304, like HU308 and HU910, was a full agonist in the hCB_2_R β-arrestin assay. However, with an EC_50_ value of 21 nM, RO6871304 was most potent. RO6871085 was a partial agonist exhibiting an efficacy value of 51%. Similar to the hCB_2_R cAMP assay, RO6851228 behaved as an inverse agonist in the hCB_2_R β-arrestin assay. None of the investigated molecules were active in the hCB_1_R β-arrestin assay. While RO6871085 and RO6851228 were not active in the mouse hCB_2_R β-arrestin assay, RO6871304 elicited a similar EC_50_ in the mouse (26 nM) and human (21 nM) CB_2_R β-arrestin recruitment. In contrast, mouse CB_2_R β-arrestin EC_50_ was lower for HU308 and HU910 (hCB_2_R β-arrestin EC_50_/mouse CB_2_R β-arrestin EC_50_ 0.2 and 0.5, respectively). Overall, the three molecules represent a tool set consisting of three highly-potent, selective, and structurally-diverse CB_2_R agonists and one potent and selective CB_2_R inverse agonist across species, which allows further pharmacological questions regarding the CB_2_R to be addressed.

### 2.3. Early Absorption, Distribution, Metabolism, Excretion, and Toxicology Profile

To assess the suitability and identify the best CB_2_R ligands for exploring *in vivo* CB_2_R pharmacology in the EIU mouse model, early ADMET data were generated (partially published for HU308 and HU910 in [28], for RO6871304 in [29], and for RO6871085 as well as RO6851228 in [32]) (Supplemental Table S1). Special emphasis was put on lipophilicity, membrane permeation properties, and stability in microsomes, as these parameters are known to influence CB_2_R responses after systemic or topical routes of administration [28]. The optimal range of molecular weight is < 500 g/mol. Where barriers such as the blood–brain barrier or the blood–retina barrier need to be crossed; molecules with calculated physicochemical values such as a topological surface area < 75 Å^2^ and a low number of hydrogen-bond donors are preferred [38,39]. The novel CB_2_R ligands meet most of these criteria. The molecular weight does not exceed 424 g/mol (cf. RO6871085). With the exception of RO6871304, for which the nitrogen atoms of the aromatic core are likely overweighed in the topological surface area calculation algorithm, all molecules have topological surface area values below 75 Å^2^. RO6871304 and RO6871085, similar to HU308 and HU910, contain one hydrogen-bond donor, while RO6851228 contains two hydrogen-bond donors. 

Lipophilicity needs to be balanced to enable membrane permeability. Experimentally generated octanol/water distribution coefficients (log*D*) are a measure for lipophilicity and look most favorable for triazolopyrimidine, RO6871304 (log*D* = 2.78). For HU910 and HU308, the calculated octanol water partition coefficient (Kow clog*P*) indicates that the molecules, like the endogenous ligands of the CB_2_R, are lipophilic. In the case of topical use for ophthalmic eye drops, lipophilic drugs can be beneficial because the contact time with the cornea is prolonged. Importantly, none of the CB_2_R ligands are charged at physiological pH since their basic pK_a_ values are all below 5. Kinetic solubility in aqueous buffer were highest for the two most polar molecules, oxetane containing pyridine, RO6851228, as well as 3-hydroxy pyrrolidine substituted triazolopyrimidine, RO6871304. The same trend was observed for thermodynamic solubilities in aqueous phosphate buffer (pH 6.5). In fasted and fed simulated intestinal fluids (FaSSIF and FeSSIF, respectively), also HU308 and HU910 exhibit high solubility (Supplemental Table S1). Importantly, all molecules are chemically stable when exposed for 2 h at 37 °C to aqueous buffers with pH values from 1 to 10.

Passive membrane permeability was assessed using the parallel artificial membrane permeability assay [40]. As the permeation coefficient *P*_eff_ is > 0.5 × 10^−6^ cm/s, all molecules partition well into the membrane and reach the acceptor compartment. RO6871304 and RO6851228 exhibit the highest permeation coefficients and reach acceptor fractions of 8% and 19%, respectively. To achieve sufficient exposure at the target tissue, low to medium clearances are desirable. Therefore, the clearance rates of the CB_2_R ligands were determined in co-incubation experiments with liver microsomes. For the triazolopyrimidine, RO6871304, and trisubstituted pyridines, RO6871085 and RO6851228, low to medium clearances in human as well as mouse microsomes were observed. The lowest clearance values (10 µL/min/kg in human microsomes and 20 µL/min/kg in mouse microsomes) were obtained for pyridine, RO6871304, which was the most polar molecule (log*D* = 2.78). Clearance data from mouse hepatocytes ranged from low (HU308) over medium (RO6871085) to high (RO6871304). In human hepatocytes, all molecules had medium clearances. Overall the clearance data from microsomes and hepatocytes were similar with the exception of HU910. For this agonist, hepatocyte clearance was lower than microsomal clearance, which may be attributed to the lower permeation coefficient. With regard to free fraction in human plasma, triazolopyrimidine RO6871304 (free fraction in human plasma 13%) and inverse agonist RO6851228 (free fraction in human plasma 9.6%) were the most beneficial. The latter molecule is; however, a strong P-gp substrate in mice (mouse P-gp-mediated efflux extraction ratios of 25.1). High penetrance through biological barriers is; therefore, more likely for the molecules with lower P-gp values, for example, 2,4,5-trisubstituted pyridine, RO6871085, which is not a P-gp substrate in human or mouse and will likely cross the brain–blood and brain–retina barriers very efficiently.

All ligands were investigated for their potential of inhibiting the most relevant cytochrome P450 isoenzymes CYP3A4, CYP2C9, and CYP2D6, and were found to show no relevant interactions at all, especially when taking their high *in vitro* potency into account. Furthermore, all ligands were clean in the glutathione (GSH) adduct formation assay, which evaluates if ligands can form reactive metabolites that could cause detrimental side effects after repeat dosing.

Data for solubility characteristics of these CB_2_R ligands in vehicles for topical, i.v., and oral delivery routes are reported in supplemental results (Supplemental Table S2). Since the route of administration used for ocular delivery in this study was topical, the plasma exposure values are assumed to be lower than that of i.v. and oral. Still, good bioavailability can be expected. Especially for the anterior ocular target tissues, the profiles of the molecules as well as the existing PK and solubility data should allow for reaching high concentrations with the evaluated ligands.

### 2.4. Immunomodulatory Properties of CB_2_R-Selective Ligands in EIU

**Figure 1:**
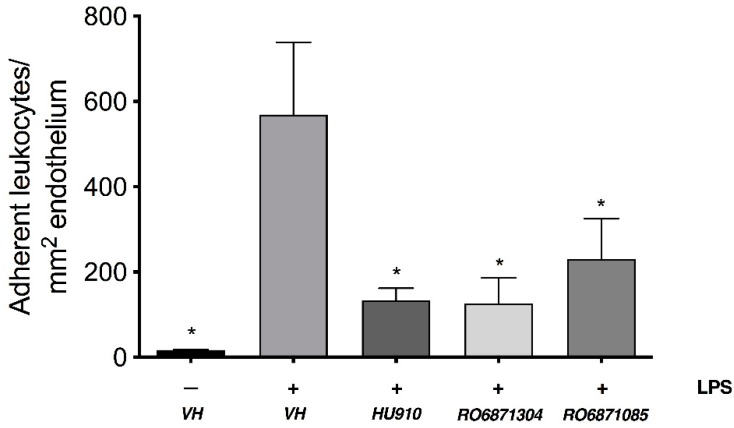
A screen of novel selective CB_2_R agonists to determine the most efficacious ligand in reducing leukocyte-endothelial adhesion in the iridial microcirculation during EIU in BALB/c mice. The bar graph represents the mean number of adherent leukocytes 6 h after intravitreal injection of: Saline (control) + topical vehicle (Tocrisolve, n = 6); lipopolysaccharide (LPS) (250 ng) + topical vehicle (n = 6); LPS + topical HU910 (1.5% *w*/*v*; n = 6); LPS + topical RO6871304 (1.5% *w*/*v*; n = 6); or LPS + topical RO6871085 (1.5% *w*/*v*; n = 6). Data are presented as mean ± SD. One-way ANOVA with Tukey’s for multiple comparisons; * *p* < 0.05 compared to LPS + vehicle.

Intravital microscopy (IVM) was carried out to evaluate the anti-inflammatory properties of these new series of selective CB_2_R agonists (RO6871304 and RO6871085) in mice with EIU. These compounds were compared to HU910, a well-established selective CB_2_R agonist based on the phytocannabinoid chemical structure backbone [20]. Figure 1 shows that intravitreal injection of LPS in wild-type (WT) animals + topical vehicle (Tocrisolve) induced a significant increase (29-fold) in the number of leukocytes adherent to the endothelium in the iridial microcirculation at 6 h compared to animals receiving an intravitreal saline injection + vehicle (*p* < 0.05). Topical treatment with 1.5% *w*/*v* HU910, RO6871304, or RO6871085 (5 µL/animal) significantly attenuated LPS-induced leukocyte-endothelial interactions at 6 h compared to vehicle-treated eyes (*p* < 0.05). There was no significant difference in the number of rolling leukocytes for any treatment group (Supplemental Table S4, *p* > 0.05). The doses used were selected based on previous studies that performed a topical dose response using the synthetic CB_2_R agonist, HU308, in rat and mouse models of EIU [23,24]. Supplemental Figure S2 shows a log dose-response with HU910 (0.03, 0.3, and 3.0 mg/kg in mixed micelles vehicle) administered i.v. directly after intravitreal LPS injection. Treatment with 3.0 mg/kg HU910 significantly reduced leukocyte adhesion in the iridial microcirculation at 6 h after LPS compared to LPS + vehicle (*p* < 0.01).

Of the CB_2_R agonists tested, RO6871304 (topical; 1.5% *w*/*v*) was the most efficacious at reducing leukocyte-endothelial adhesion in the iridial microcirculation at 6 h following induction of EIU. RO6871304 also had the highest selectivity for the CB_2_R over the CB_1_R (Table 2). Therefore, this compound was used for subsequent studies examining the CB_2_R therapeutic time-window, CB_2_R effects on neutrophil migration, and whether selective CB_2_R agonists act on resident ocular immune cells.

**Figure 2:**
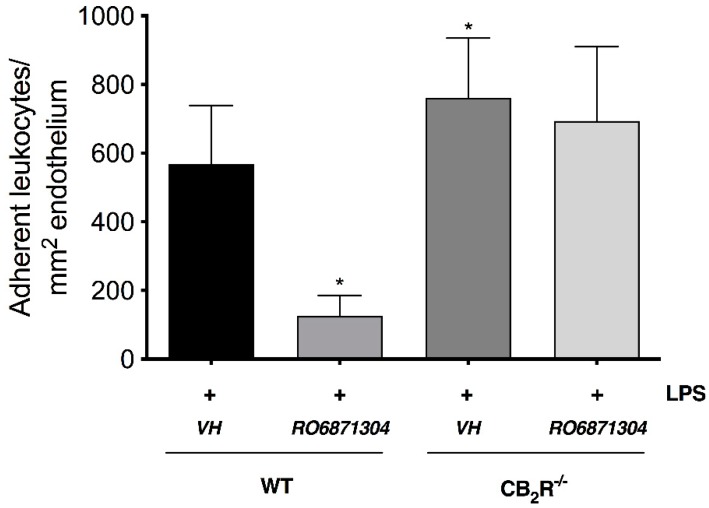
RO6871304 exerts its anti-inflammatory effects through CB_2_R. CB_2_R^-/-^ mice with EIU exhibit increased iridial leukocyte-endothelial adhesion compared to wild-type (WT). Bar graph represents the mean number of adherent leukocytes in WT or CB_2_R^-/-^ mice 6 h following intravitreal injection of LPS and topical treatment with either vehicle (Tocrisolve, n = 7) or RO6871304 (1.5% *w*/*v*; n = 6). Data are presented as mean ± SD. One-way ANOVA with Dunnett; * *p* < 0.05 compared to LPS + vehicle in WT mice.

**Figure 3:**
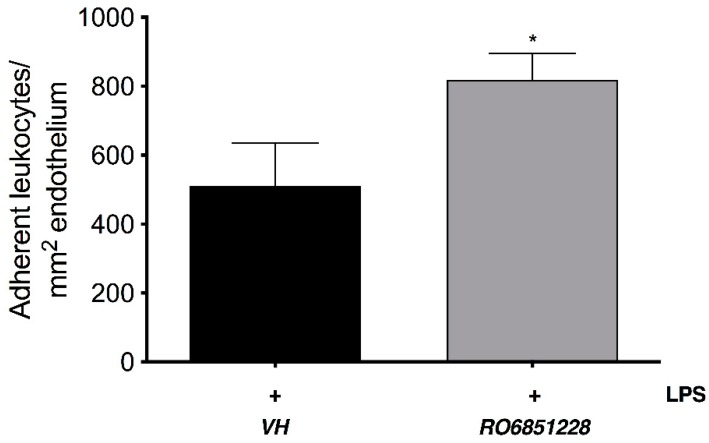
The CB_2_R inverse agonist, RO6851228 increases leukocyte adhesion in the iridial microcirculation during EIU. Bar graph represents the mean number of adherent leukocytes at 6 h post-intravitreal injection of LPS and following topical treatment with either vehicle (Tocrisolve, n = 8) or RO6871228 (1.5% *w*/*v*; n = 6). Data are presented as mean ± SD. Two-tailed unpaired t-test; * *p* < 0.05 compared to LPS + vehicle.

To further examine the involvement of CB_2_R in the EIU pathology and to validate CB_2_R as the target for these new selective CB_2_R agonists *in vivo*, CB_2_R^-/-^ mice were used. Figure 2 shows that LPS-induced inflammation in CB_2_R^-/-^ mice + vehicle was significantly increased relative to WT + LPS-injected + vehicle animals (*p* < 0.05). In contrast to WT mice, there was no significant reduction in leukocyte adherence in CB_2_R^-/-^ mice treated with RO6871304 (1.5% *w*/*v*). Consistent with this, Figure 3 shows that treatment of WT mice with the selective CB_2_R inverse agonist RO6851228 (1.5% *w*/*v*) increased leukocyte-adhesion at 6 h post-intravitreal injection of LPS compared to treatment with vehicle.

### 2.5. Effects of CB_2_R-Selective Ligands on Neutrophil Migration In Vitro

**Figure 4:**
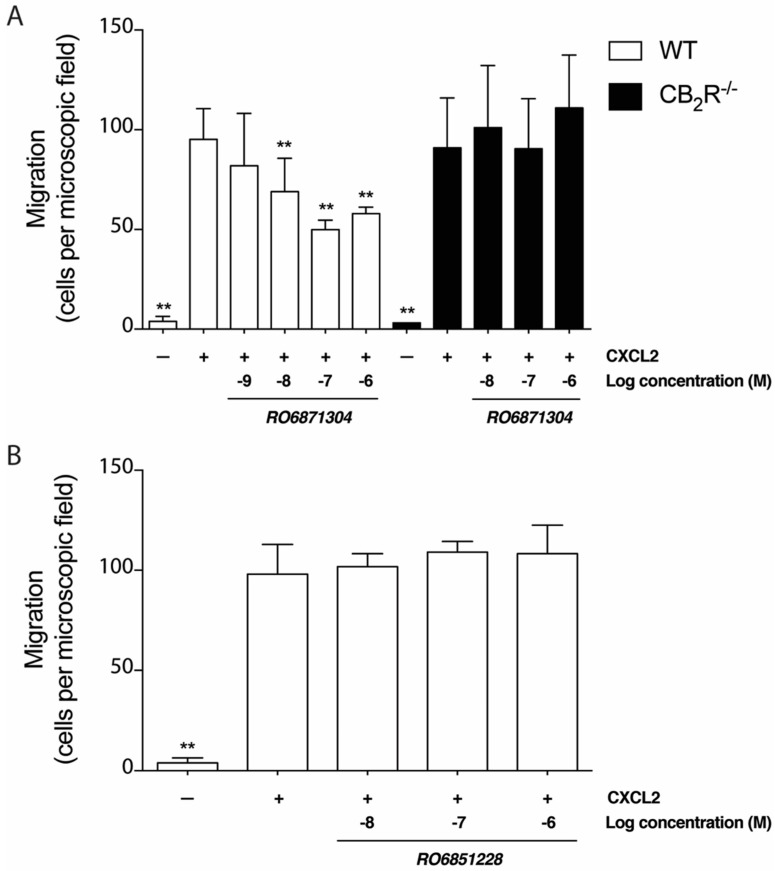
CB_2_R activation reduces neutrophil migration towards chemoattractant. (**A**) Bar graph represents the mean number of neutrophils from WT or CB_2_R^-/-^ mice pre-treated with various concentrations of the CB_2_R agonist, RO6871304, that migrated to the lower chamber of a Boyden chamber setup when using CXCL2 (10^−8^ M) as a chemoattractant. Neutrophils were counted from 12 random microscopic fields of view per well. (**B**) Bar graph represents the mean number of neutrophils from WT mice pre-treated with various concentrations of the CB_2_R inverse agonist, RO6851228, or vehicle (0.01% DMSO) that migrated in the Boyden chamber setup. Data are presented as the mean neutrophil migration per microscopic field of view ± SD from at least three independent experiments conducted in triplicate each. One-way ANOVA with Dunnett; ** *p* < 0.01 compared to WT neutrophils + CXCL2 + vehicle.

To determine whether the CB_2_R agonist, RO6871304, modulates neutrophil migration towards a chemoattractant signal, a Transwell migration assay was performed with CXCL2 placed in the lower chamber. CXCL2 is a potent neutrophil chemoattractant [41]. As expected, basal migration of mouse neutrophils was minimal, but increased significantly in the presence of 10 nM CXCL2 (Figure 4A). RO6871304 inhibited neutrophil migration towards CXCL2 in a dose-dependent fashion (Figure 4A). The IC_50_ for RO6871304-mediated inhibition of neutrophil migration was 6.6 nM. CB_2_R^-/-^ neutrophils migrated toward CXCL2 as efficiently as WT cells (Figure 4A). However, RO6871304 did not inhibit migration of CB_2_R^-/-^ neutrophils (Figure 4A). Together, these data demonstrate that RO6871304 inhibits CXCL2-induced chemotaxis of primary mouse neutrophils in a concentration-dependent manner dependent on the presence of CB_2_R.

Neutrophils were treated with RO6851228 to determine the effect of this CB_2_R inverse agonist on migration. Treatment of neutrophils with RO6851228 (0.01–1.0 μM) did not affect CXCL2-induced neutrophil migration compared to vehicle (Figure 4B), suggesting that neutrophil migration is already at its maximal in this system or that there is no constitutive CB_2_R activity in this *in vitro* system. 

### 2.6. Adoptive Transfer

#### CB_2_R Activation on Resident Immune Cells Mediates CB_2_R Leukocyte Adherence Effects

To determine the contribution of resident ocular immune cells to the CB_2_R-mediated decrease in leukocyte adhesion in the iridial microcirculation seen after CB_2_R agonist treatment (RO6871304 and RO6871085), donor bone-marrow leukocytes from naïve mice were adoptively transferred at 5 h post-EIU. Treatment with Gr-1 antibody (50 μg in PBS; i.p.) decreased circulating neutrophils in the peripheral blood of host mice after 24 h compared to isotype control, as determined by fluorescence-activated-cell sorting (FACS) analysis of peripheral blood for Gr-1^+^ (Supplemental Figure S3). As expected, neutrophil depletion in EIU mice blocked LPS-induced leukocyte (autologous) adhesion in the iridial microcirculation (Supplemental Figure S4), confirming that neutrophils are the primary cells adhering to the inflamed iridial endothelium. 

**Figure 5:**
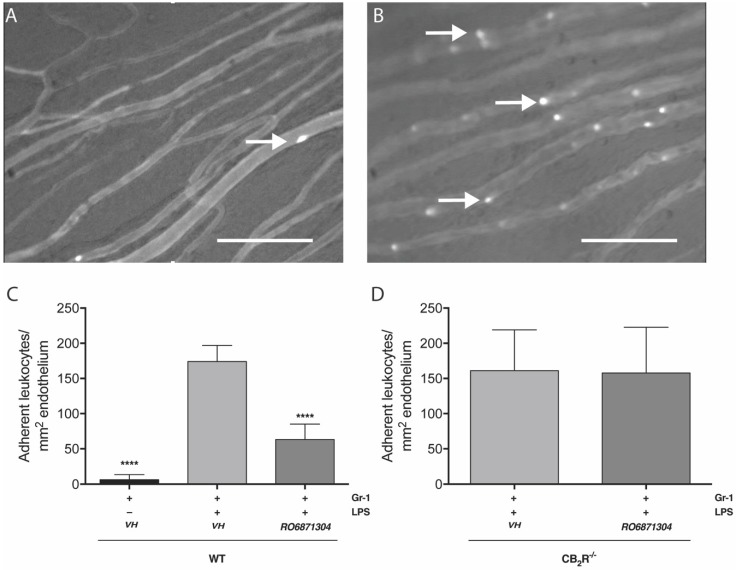
CB_2_R activation in neutrophil-depleted EIU mice with accompanying leukocyte adoptive transfer reduces leukocyte adhesion in the iridial microcirculation. Representative intravital microscopy (IVM) images of the iridial microcirculation of neutrophil-depleted mice showing the number of adoptively-transferred adherent leukocytes [calcein-acetoxymethyl ester (AM) pre-labeled] 6 h after intravitreal saline (**A**) or LPS (**B**). Arrows indicate adherent leukocytes. Scale bar = 100 μm. (**C**) bar graph represents the mean number of adoptively-transferred WT leukocytes adherent to the iris microcirculation 6 h after EIU induction with LPS injection or saline control in neutrophil-depleted WT mice treated topically with vehicle (Tocrisolve) or RO6871304 (1.5% *w*/*v*). n = 6-8; one-way ANOVA with Dunnett; **** *p* < 0.0001 compared to LPS + vehicle. (**D**) bar graph represents the mean number of adoptively-transferred WT leukocytes adherent to the iris microcirculation 6 h after EIU in neutrophil-depleted CB_2_R^-/-^ mice treated topically with vehicle (Tocrisolve) or RO6871304. n = 6–8; two-tailed unpaired t-test; **** *p* > 0.05 compared to LPS + vehicle.

Figure 5A,B shows representative images of the iris microcirculation 1 h after adoptive transfer of naïve bone-marrow derived leukocytes into neutrophil-depleted mice that were treated 5 h previously with saline (control) or LPS (EIU). Figure 5 shows that at 6 h post-EIU in neutrophil depleted mice, there was a significant increase (28-fold) in the number of adoptively-transferred leukocytes adherent to the iridial microcirculation compared to animals receiving intravitreal saline control. Topical treatment with the CB_2_R agonist, RO6871304 (Figure 5C; 1.5% *w*/*v*), in neutrophil-depleted mice with EIU significantly decreased (64-fold) the LPS-induced adhesion of adoptively-transferred leukocytes compared to vehicle. There was no difference in the number of WT adoptively-transferred leukocytes adherent to the iridial endothelium in neutrophil-depleted WT recipients with EIU compared to neutrophil-depleted CB_2_R^-/-^ recipients with EIU. Furthermore, RO6871304 treatment did not affect adhesion of adoptively-transferred leukocytes (Figure 5D), suggesting that RO6871304 is acting primarily on resident cells in the eye to alter leukocyte adhesion.

## 3. Discussion

This study investigated the activity of novel cannabinoids that are highly potent and selective for the CB_2_R over CB_1_R, with the goal of identifying ligands with good drug-like properties that can be used as tool/lead compounds to further validate CB_2_R as a drug target for ocular inflammatory disease. The compounds RO6871304, RO6871085, and RO6851228 were chosen for investigation because they have an overall improved drug-likeness/ADMET profile compared to other published cannabinoids (e.g., HU308 and HU910). RO6871304, RO6871085, and RO6851228 have decreased lipophilicity compared to other published cannabinoids (e.g., HU308 and HU910), good solubility, passive membrane permeability, and low microsomal clearance. Our studies demonstrate that topical application to the cornea of these ligands is efficacious in attenuating leukocyte adhesion to the iridial microvasculature in EIU, congruent with anti-inflammatory actions. Studies of neutrophil migration *in vitro* indicated that, while selective CB_2_R agonists inhibit neutrophil migration, the effects of these ligands *in vivo* may be primarily mediated via actions on resident ocular immune cells. 

Panuveitis affects tissues in the anterior chamber of the eye, including the iris [42]. Therefore, we took advantage of IVM to non-invasively observe and quantify the effects of CB_2_R agonists on the behavior of leukocytes in the iridial microvasculature in live animals with LPS-induced EIU. LPS increases leukocyte-endothelial interactions in the iridial microcirculation, a step which precedes the transendothelial migration of leukocyte into the affected tissue [16]. Our goal was to identify ligands that were potent and selective for CB_2_R and devoid of potential off-target effects, and to explore the potential of these ligands for the treatment of LPS-induced EIU. Off-target activity could arise due to actions at other receptors, including CB_1_R as well as other non-cannabinoid receptors, or activity at enzymes involved in endocannabinoid regulation or direct effects on ion channels [27,28,43]. Our competitive ligand binding assays and functional assays demonstrated that RO6871304, RO687108, and RO6851228 are highly selective for CB_2_R over CB_1_R. RO6871304 and RO6871085 displayed anti-inflammatory efficacy comparable or better than the well-characterized CB_2_R-selective agonists, HU910 and HU308. In EIU, topical treatment of animals with RO6871304, RO6871085, like HU910, decreased adhesion of leukocytes to the endothelium. Topical drug delivery was the preferred route of administration, as it avoids systemic effects and is the most commonly used method for applying ophthalmic medications [44]. As RO6871304 was the most efficacious of the ligands tested in reducing leukocyte adhesion and exhibited the highest selectivity *in vitro* for the human CB_2_R over CB_1_R, this cannabinoid was used for the majority of the pharmacological studies including validation of target. We showed that the anti-inflammatory effect of RO6871304 was selective for the CB_2_R and its effect was absent in CB_2_R^-/-^ animals with EIU.

CB_2_R has been reported to be upregulated in inflammation [18,45,46,47,48]. Work from our laboratory using a mouse model of EIU indicated that CB_2_R mRNA in ocular tissue is increased, [24] and that constitutive activity and/or increased endogenous activation of CB_2_R may modulate the ensuing inflammatory response to endotoxin [23,24]. Work presented here further supports the findings of Toguri et al. (2014 and 2018), and indicates that CB_2_R upregulation leads to increased activity of this receptor in EIU, as the inverse agonist, RO6851228, increased leukocyte adhesion in mice with EIU. Systemic administration of a CB_2_R inverse agonist administered prior to endotoxin induction of sepsis in mice was reported to enhance inflammatory damage [49]. In models of proliferative vitreoretinopathy and EIU [22,23], systemic and topical administration of CB_2_R antagonist/inverse agonists increased inflammation. Our results using the inverse agonist, RO6851228, are consistent with these studies and further imply that ocular inflammation results in upregulation of CB_2_R in ocular tissues. Furthermore, we also found that CB_2_R^-/-^ mice exhibited increased leukocyte adhesion during EIU. Taken together, this suggests that there is increased constitutive activity of CB_2_R during ocular inflammation, which may contribute to an auto-protective role of this receptor in ocular pathology. 

We also examined immune cell targets and mechanisms that may contribute to the immunosuppressive actions of CB_2_R activation. Our work demonstrated that the selective CB_2_R agonist, RO6871304, concentration-dependently inhibited neutrophil migration toward the chemoattractant, CXCL2. However, pre-treatment with the inverse agonist, RO6851228, had no effect on neutrophil migration compared to vehicle in neutrophil migration assays. The lack of expected pro-migratory activity following treatment with the inverse agonist, RO6851228, could be due to several factors. For example, the maximal number of neutrophils able to migrate was already reached, and/or or neutrophils do not exhibit constitutive CB_2_R activity, at least in this *in vitro* assay system. 

CB_2_R activation has been shown to reduce leukocyte adhesion using IVM in several studies [16,49,50,51]; however, these studies did not investigate the cellular targets (resident immune cells, circulating neutrophils, or endothelium) responsible for this effect. This is because the IVM approach used for these experiments does not easily allow discrimination between fluorescent-labelled individual immune cell subtypes. Therefore, to obtain further information on specific immune cells involved in the inflammatory response in EIU, we adopted an alternative method to help distinguish specific cell types mediating the anti-inflammatory effects of CB_2_R activation. The approach we used was to selectively deplete circulating neutrophils prior to the induction of EIU, and subsequently treat eyes topically with a CB_2_R agonist before adoptive transfer of leukocytes. The results showed that treatment of eyes with a CB_2_R agonist, RO6871304, significantly inhibited adhesion of adoptively-transferred leukocytes compared to vehicle-treated eyes. This implies that the effects of RO6871304, are largely exerted via binding to CB_2_R on the ocular resident immune cells (macrophages and/or microglia) and/or endothelium rather than neutrophils, as RO6571304 did not reduce adhesion of WT leukocytes transferred into CB_2_R^-/-^ mice.

Our results demonstrate that novel cannabinoid ligands, which selectively bind and activate CB_2_R, attenuate leukocyte adhesion to the iridial microvasculature in EIU, an effect which is consistent with the anti-inflammatory role for this receptor. From the research carried out in this study, we propose that these ligands exert their effects at multiple immune cell targets to decrease inflammation. These include neutrophils, since pre-treatment of neutrophils with these ligands decreases their migration *in vitro* toward CXCL2, and resident ocular cells (microglia, monocytes, and/or endothelium), given that CB_2_R-selective ligands reduced adhesion of adoptively-transferred leukocytes in neutrophil-depleted EIU mice. Our results strongly suggest that drugs targeting CB_2_R may have efficacy in the treatment of uveitis by decreasing neutrophil recruitment, activation of resident ocular immune cells and/or endothelium, and preventing consequent tissue damage. The ability to deliver these drugs topically, as well as via other routes of delivery, suggests that they may be used to treat ocular inflammation originating in both anterior or posterior ocular tissues.

## 4. Materials and Methods

### 4.1. Cell Culture and Membrane Preparations

Membrane fractions were prepared from cultured CHOK1hCB_1__bgal, CHOK1hCB_2__bgal, and CHOK1mCB_2__bgal cells (DiscoveRx, Fremont, CA, USA) as previously described [28]. Protein concentration was determined using the Pierce bicinchoninic acid (BCA) protein assay (Rochford, IL, USA) as per the manufacturer’s instructions.

### 4.2. Mouse CB_2_R and Human CB_2_R and CB_1_R Binding Assays

Radio ligand-binding assays were carried out as described by Souethoudt et al. (2018) [28]. Briefly, membranes from cells expressing human CB_2_R or CB_1_R or mouse CB_2_R and the radioligand, [^3^H]-CP55940 (Perkin Elmer, Basel, Switzerland) was used. K_i_ values were calculated from a single experiment using triplicates of 10 different concentrations of compound [37]. Membrane aliquots containing 5 µg (CHOK1hCB_1__bgal) or 1 µg (CHOK1hCB_2__bgal) of membrane protein in 100 µL assay buffer (50 mMTris-HCl, 5 mM MgCl_2_, 0.1% bovine serum albumin (BSA), pH 7.4) were incubated at 30 °C for 1 h, in presence of 3.5 nM of [^3^H]-CP55940 (CHOK1hCB_1__bgal) or 1.5 nM [^3^H]-CP55940 (CHOK1hCB_2__bgal). The incubation was terminated by rapid filtration performed on glass microfibre (GF/C) filters (Whatman International, Maidstone, UK), presoaked for 30 min with 0.25% polyethyleneimine (PEI), and using a Brandel harvester (Brandel, Gaithersburg, MD, USA). Filter-bound radioactivity was determined by scintillation spectrometry using a Tri-Carb 2900 TR liquid scintillation counter (Perkin Elmer, Boston, MA, USA). For mouse experiments, spleens were collected as previously described [28] and resuspended in 2 mM Tris-EDTA, 320 mM sucrose, 5 mM MgCl_2_ (pH 7.4). Tissue was homogenized with a Potter homogenizer and centrifuged three times at 1000× *g* (10 min). The supernatants were centrifuged at 18,000× *g* (30 min), and the pellets were resuspended in assay buffer (50 mM Tris-HCl, 2 mM Tris-EDTA, 3 mM MgCl_2_, pH 7.4). These membrane fractions were used in rapid filtration assays with 400 pM of [^3^H]-CP55,940. In all receptor binding experiments, nonspecific binding was determined in the presence of 1 µM “cold” agonist. 

### 4.3. PathHunter β-Arrestin Recruitment Assays

Assays were carried out using the PathHunter hCB_1__bgal, hCB_2__bgal, or mCB_2__bgal CHOK1 β-arrestin recruitment assay kit (DiscoveRx, Fremont, CA, USA) as previously described [28]. A total of 5000 cells per well were seeded in 384-well plates (Perkin Elmer, MA, USA) containing 20 μL cell culture medium and incubated for 16–18 h at 37 °C and 5% CO_2_. Cells were stimulated with 10 μM of each agonist or 11 increasing concentrations and incubated for 90 min at 37 °C and 5% CO_2_. In the inverse agonist assays, cells were exposed to 10 μM of RO6851228 or 11 increasing concentrations and incubated for 30 min at 37 °C and 5% CO_2_, followed by the addition of the EC_80_ concentration of CP55940 (25 nM for CHOK1hCB_1__bgal and 46 nM for CHOK1hCB_2__bgal). The cells were incubated for 90 min at 37 °C and 5% CO_2_. Compounds in DMSO stock solutions were added using a HP D300 Digital Dispenser (Tecan, Mannedorf, Switzerland). The final concentration of organic solvent per assay point was ≤ 0.1%. The PathHunter Detection mixture was used, according to the manufacturer’s protocol, to determine the β-galactosidase enzyme activity. Detection mixture, 12 μL per well, was added and the plate was incubated for 1 h in the dark at room temperature. Chemiluminescence, indicated as relative light unit, was measured on an EnVision multilabel plate reader (Perkin Elmer, MA, USA).

### 4.4. cAMP Assays

The effect of ligands on the forskolin-stimulated accumulation of cAMP was determined using the LANCE ULTRA cAMP kit as per the manufacturer’s instruction (Perkin-Elmer Life Sciences, Boston, MA, USA). 

For mouse CB_2_R cAMP assays, mouse spleen was resuspended in 50 mM of Tris-HCl (pH 7.4), then homogenized with a Potter homogenizer and centrifuged at 1000× *g* for 10 min. The supernatants were incubated for 30 min with 1-methyl-3-isobutylxanthine (IBMX), then forskolin was added in the presence or absence of ligands. The supernatants were incubated for 30 min in ATP Regeneration Buffer (50 mM HEPES, pH 7.4, 10 mM phosphocreatine, 10 units/mL creatine phosphokinase, 10 μM GTP, 200 μM ATP, 10 mM MgCl_2_, 250 μM IBMX), and the reaction was stopped by adding lysis buffer. Time-resolved fluorescence was measured with a Victor V Multilabel counter (Perkin-Elmer Life Sciences, Boston, MA, USA). 

For human CB_2_R and CB_1_R, cAMP assays were performed using CHO cells stably expressing human CB_2_R or human CB_1_R (DiscoveRx, Fremont, CA, USA) using a cAMP-Nano-TRF detection kit (Roche Diagnostics, Penzberg, Germany). Cells were seeded 17–24 h prior to the experiment at a density of 3 × 10^4^ cells per well in a black 96-well plate with clear flat bottom (Corning, Wiesbaden, Germany) and incubated in 5% CO_2_ at 37 °C in a humidified incubator. The growth medium was exchanged with Krebs Ringer bicarbonate buffer with 1 mmol/L 3-isobutyl-1-methylxanthine (IBMX), 0.1% fatty acid-free BSA and incubated at 30 °C for 60 min. Agonist was added to a final assay volume of 100 μL and the mixture was incubated for 30 min at 30 °C. The assay was stopped by the addition of 50 μL 3 × lysis reagent and shaken for 2 h at room temperature. The time-resolved fluorescence energy transfer was measured using an LF502 Nanoscan FLT (IOM, Berlin, Germany), equipped with a laser as excitation source. cAMP content was determined from the function of a standard curve spanning from 10 to 0.13 nmol/L cAMP. Efficacies are expressed as percent relative to 1 µM CP55940. EC_50_ values are the average of determinations (n = 1) performed in triplicate.

### 4.5. Animals

Male BALB/c wild-type (WT; 25-30 g; Charles River, QC, Canada) and age-matched (6–8 weeks) CB_2_R knockout mice (CB_2_R^-/-^) were used for experiments. CB_2_R^-/-^ mice were obtained by crossing male C57BL/6J CB_2_R^-/-^ mice (strain B6.129P2-Cnr2tm1Dgen/J; Jackson Laboratory, Bar Harbor, ME, USA) with inbred BALB/c female mice (Charles River) for ten generations. Heterozygote mice from separate parents were then bred for a homozygote knock-out of the CB_2_R^-/-^. CB_2_R^-/-^ was confirmed via genotyping using DNA extracted from ear punches and the Accustart II Mouse Genotyping Kit (Quanta Biosciences, MD, USA). The following PCR primers were used: moIMR0086 (5′-GGGGATCGATCCGTCCTGTAAGTCT-3′; mutant forward), oIMR7552 (5′-GACTAGAGCTTTGTAGGTAGGCGGG-3′; common reverse), and oIMR7565 (5′-GGAGTTCAACCCCATGAAGGAGTAC-3′; WT forward). The expected results were a single product at ~550 bp for CB_2_R^-/-^, a single product at ~385 bp for wild-type, and both products for heterozygous animals [24].

BALB/c mice were used for intravital microscopy (IVM) experiments as the absence of pigment allowed for visualization of leukocyte-endothelial interactions in the iridial microvasculature [24]. Animals were maintained on a 12 h light–dark cycle with unrestricted access to food and water. All experiments were conducted in accordance with the standards and procedures of the Canadian Council on Animal Care (www.ccac.ca) and were approved by the Dalhousie University Committee on Laboratory Animals (Protocols 14-111 and 16-066). All studies involving animals are reported in accordance with the ARRIVE guidelines for reporting experiments involving animals [52,53].

### 4.6. Endotoxin-Induced Uveitis (EIU) Model

EIU was induced in BALB/c mice using an intravitreal injection of LPS (2 μL; 125 ng/μL; *E. coli* 0111:B4 L084M4118V; Sigma-Aldrich, Oakville, ON, Canada) in saline [23,42],. Animals were anesthetized with isoflurane (4% induction, 2% maintenance), and were injected intravitreally through the pars plana with sterile saline (control) or LPS solution using a 30G needle and Hamilton syringe (Hamilton Company, Reno, NV, USA) with the assistance of a WILD M37 dissecting microscope (Leitz Canada, Kitchener, ON, Canada). The puncture wound was glued shut with 3M Vetbond Tissue Adhesive (3M Animal Products, St. Paul, MN, USA), and animals were allowed to recover.

### 4.7. Intravital Microscopy (IVM)

IVM was conducted as previously described in Toguri et al. (2014). The iridial microcirculation was imaged 6 h after induction of EIU. Animals were anesthetized with pentobarbital (i.p., 54 mg/kg). Fifteen minutes before initiating IVM, 0.05% Rhodamine 6G (1.5 mL/kg; Sigma-Aldrich) was injected intravenously (i.v.) to visualize leukocytes within the vasculature, while 5% fluorescein isothiocyanate (FITC) conjugated albumin (i.v., 1 mL/kg; Sigma-Aldrich) was used to visualize blood flow. The iridial microcirculation was observed using an Olympus OV100 Small Animal Imaging System (Olympus, Tokyo, Japan), which contains an MT-20 light source, fluorescence excitation from a xenon lamp (150 W) and an excitation filter for rhodamine-6G (excitation 515–560 nm, emission 590 nm). Images were captured in real-time by a black and white DP70 CCD C-mount camera and digitally recorded using HCImage software (Hamamatsu, Herrsching, Germany).

For adoptive transfer experiments, calcein-AM-labelled leukocytes were visualized by fluorescence (495 nm excitation) in the iridial microcirculation using a Leica DMLM epifluorescence microscope (Leica, Wetzlar, Germany) equipped with a mercury-arc light source (LEG EBQ 100, Carl Zeiss, Jena, Germany) and captured by a CCD video camera (SIT 68, DAGE MTI, Michigan, IN, USA). Videos were recorded using WinDV Capture software (Mourek, Prague, Czech Republic) and stored on an external hard drive. 

During IVM animals were placed on a heating pad in a stereotactic frame, and Tear-Gel^®^ (Novartis Pharmaceuticals Canada Inc., Dorval, QC, Canada) was applied to their cornea. The iris was divided into four equal quadrants by drawing two superficial lines on the cornea, lengthwise and widthwise. IVM was carried out in each of these quadrants. Two to four videos of each quadrant were recorded for 30 s each. Videos were analyzed off-line without knowledge of the treatment groups. Adherent leukocytes were defined as the number of leukocytes that did not detach from the endothelial surface during the 30 s observation period in venules. Using ImageJ software (National Institute of Health, Bethesda, MD, USA) the number of adherent leukocytes within each vessel segment was calculated by measuring the diameter and length of vessel segment studied, assuming a cylindrical geometry of blood vessels. Adherent leukocytes were expressed as number of cells per mm^2^ of endothelial surface. Rolling leukocytes were classified as cells that moved along the endothelium and crossed a predetermined cross-sectional line of the venule. The number of rolling leukocytes was counted for 30 s and used to calculate the number of rolling leukocytes per min. IVM was conducted 6 h after induction of EIU.

### 4.8. Isolation of Murine Bone Marrow Cell Neutrophils

As described previously [54], mouse neutrophils were isolated from the bone marrow of mice. Mice were euthanized, femur and tibia were dissected and flushed with ice-cold 1× Hanks balanced salt solution (HBSS; supplemented with 10 nM HEPES, pH7.5, and 0.5 mM EDTA; Invitrogen) through a sterile filter (70 μm). The suspension was centrifuged at 300× *g* for 12 min at 4 °C. EasySep™ neutrophil enrichment kit (STEMCELL Technologies Inc., Vancouver, BC, Canada) was used to isolate neutrophils according to the manufacturer’s instructions. The isolated neutrophils were then washed once and the pelleted cells were suspended in RPMI (Invitrogen) containing 1% heat-inactivated fetal bovine serum (FBS, Invitrogen) and used immediately for neutrophil migration experiments or processed for adoptive transfer. The murine neutrophil isolation protocol routinely yields cell suspensions that are >95% pure and viable, as determined by cresyl violet and trypan blue exclusion, respectively.

### 4.9. Neutrophil Migration Assay

Neutrophil migration assays were performed using a Transwell migration assay [55,56]. Isolated neutrophils (1 × 10^5^ cells/100 μL) from WT or CB_2_R^-/-^ mice were incubated with vehicle (0.01% DMSO), or various concentrations of RO8671304 or RO6851228 at 37 °C, 5% CO_2_ for 30 min. Cells were then loaded into the upper chamber of 3 μm Transwell inserts (Corning Life Sciences, Corning, NY, USA) coated with 5% FBS. Transwells were placed in 24-well plates containing RPMI (1% FBS) alone or 10^−8^ M chemokine C–X–C motif ligand 2 (CXCL2)/monocyte inflammatory protein-2 (Peprotech, Rocky Hill, NJ, USA). Plates were incubated for 1 h at 37 °C, 5% CO_2_. To determine the number of neutrophils that migrated from the top to the bottom chamber, the filter was removed and neutrophils in the lower chamber gently pipetted to evenly distribute and sink to the bottom of the pate. An Olympus inverted bright-field microscope with an Infinity 3 camera (Lumenera, Ottawa, ON, Canada) was used to photograph 12 random fields (10× magnification) of each well. The number of migrated neutrophils was then determined using ImageJ. The data represent the mean value of cells per field ± standard deviation from at least three independent experiments for each treatment condition performed in triplicate.

### 4.10. Neutrophil-Depletion and Adoptive Transfer

Neutrophils were depleted *in vivo* using mAb Gr-1 (RB6-8C5, BioXcell, West Lebanon, NH, USA) administered 24 h prior to induction of EIU, as previously described [57]. Mice were injected with a single dose of 50 μg in 100 μL Gr-1 (i.p.) or isotype control antibody (IgG2b; BioXcell). Treatment with this dose of antibody induced severe neutropenia for up to 48 h. EIU was induced 24 h after Gr-1 treatment. Directly after induction of EIU, mice were treated with vehicle or the CB_2_R agonist, RO6871304, topically (5 µL; 1.5% w/v) to the cornea of the left eye. Leukocytes isolated from mouse bone marrow were stained with 1 μM calcein-AM (Life Technologies, Gaithersburg, MD, USA) at 37 °C, 5% CO_2_ for 30 min, washed and resuspended in PBS for injection. Five hours after induction of EIU, neutrophil-depleted (Gr-1 treated) animals were anesthetized and adoptively-transferred i.v. with 1.5 × 10^7^ calcein-AM pre-loaded leukocytes and allowed to circulate for 1 h prior to imaging. 5% FITC (i.v., 1 m/kg) was injected 10 min before IVM.

### 4.11. Peripheral Blood Leukocyte Isolation

To characterize the efficiency of neutrophil depletion using Gr-1-mediated antibody depletion, blood was obtained 24 h following injection of anti-Gr-1. Submandibular venipuncture was performed to collect 70 μL of blood in 10 μL of heparin (10,000 U/mL; Sigma-Aldrich). Red blood cell lysis was performed by adding 2 mL of lysis buffer for 1 min and neutralized by adding an equal volume of PBS. Samples were centrifuged for 5 min at 300 g, supernatant was discarded and the blood leukocytes were resuspended in 200 μL of PBS. The frequency of Gr-1^+^ CD11b^+^ cells was determined as described below using flow cytometry [58].

### 4.12. Antibodies and Flow Cytometry

The following antibodies were obtained from eBioscience (San Diego, CA): FITC-conjugated rat IgG2b (clone A95-1), FITC-anti-CD11b (clone M1/70); phycoerythrin-conjugated rat IgG2b (clone A95-1), phycoerythrin-anti-Gr1 (clone RB6-8C5). Prior to staining, all cell samples were pre-incubated with anti-CD16/32 to block non-specific binding. Flow cytometry was carried out using a two laser FACSCalibur with BD CellQuest Pro software (BD Biosciences, Mississauga, ON, Canada) and data analysis was performed using FlowJo (V10.2; FlowJo, LLC; Ashland, OR).

### 4.13. Drug Treatments

CB_2_R agonists, RO6871304 [33], RO6871085 [30], and the CB_2_R inverse agonist, RO6851228 [32], were provided by Hoffman-la Roche Ltd. (Basel, Switzerland), and were dissolved for topical administration in Tocrisolve™ 100 (Tocris Bioscience, Ellisville, MO, USA), which is a 1:4 ratio of soya oil:water, that is emulsified with the block co-polymer Pluronic F68. RO6871304, RO6871085, or RO6851228 were applied topically on the left eye (1.5% *w*/*v*; 5 μL/animal)) directly after injection of LPS in the same eye. Control animals received vehicle (Tocrisolve) (5 μL/animal) only. HU910 was dissolved for i.v. administration in mixed micelles.

### 4.14. Statistical Analysis

Individual animals in each treatment group were coded and experiments were analyzed blinded. All data are expressed as mean ± standard deviation, and results were analyzed using Prism 5 software (GraphPad Software, La Jolla, CA, USA). The Kolmogorov–Smirnov test was used to confirm normal distribution of the data. A two-tailed unpaired t-test was used to compare two groups of data. One-way analysis of variance with a Dunnett’s or Tukey’s post hoc test was used to compare multiple data groups. Significance was set at *p* < 0.05.

## Figures and Tables

**Table 1:** Chemical structures and International Union of Pure and Applied Chemistry (IUPAC) names of cannabinoid 2 receptor (CB_2_R) ligands used in EIU model.

Compound	HU-308	HU-910	RO6871304	RO6871085	RO6851228
**Chemical structure**	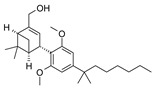	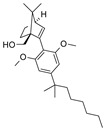	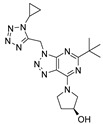	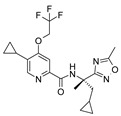	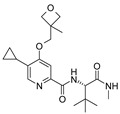
**IUPAC name**	{(1*S*,4*S*,5*S*)-4-[2,6-Dimethoxy-4-(2-methyloctan-2-yl)phenyl]-6,6-dimethylbicyclo[3.1.1]hept-2-en-2-yl}methanol	{(1*S*,4*R*)-2-[2,6-dimethoxy-4-(2-methyloctan-2-yl)phenyl]-7,7-dimethylbicyclo[2.2.1]hept-2-en-1-yl}methanol	(3*S*)-1-{5-tert-butyl-3-[(1-cyclopropyl-1H-tetrazol-5-yl)methyl]-3H-[1,2,3]triazolo[4,5-d]pyrimidin-7-yl}pyrrolidin-3-ol	5-Cyclopropyl-*N*-[(2*S*)-1-cyclopropyl-2-(5-methyl-1,2,4-oxadiazol-3-yl)propan-2-yl]-4-(2,2,2-trifluoroethoxy)pyridine-2-carboxamide	5-Cyclopropyl-*N*-[(2*S*)-3,3-dimethyl-1-(methylamino)-1-oxobutan-2-yl]-4-[(3-methyloxetan-3-yl)methoxy]pyridine-2-carboxamide

**Table 2:** *In vitro* pharmacology data for cannabinoid 2 receptor (CB_2_R) agonists HU308, HU910, RO6871304, RO6871085, and CB_2_R inverse agonist RO6851228. (These data have been generated at the Roche Laboratories and were partially published, see also [28,29,32]). Due to their relevance for the described pharmacology studies, they are provided here in a condensed format. * Indicates previously published data.

Compound	HU308	HU910	RO6871304	RO6871085	RO6851228
hCB_2_R: Ki (nM)	10 *	43 *	17	76 *	45 *
hCB_1_R: Ki (nM)	4600 *	3734 *	>10,000 *	3378 *	6487 *
hCB_1_R Ki/hCB_2_R Ki	460	89	>588 *	44 *	147 *
mouse CB_2_R: Ki (nM)	223	276	34	6 *	1 *
hCB_2_R: cAMP EC_50_ (nM), efficacy (%)	3, 98 *	4, 98 *	1, 102 *	8, 88 *	26, −159 *
hCB_1_R: cAMP EC_50_ (nM)	>10,000 *	>10,000 *	>10,000 *	1449, 38 *	>10,000
hCB_1_R cAMP EC_50_/hCB_2_R cAMP EC_50_	>3333	>2500	>10,000	174 *	>383 *
mouse CB_2_R: cAMP EC_50_ (nM), efficacy (%)	6, 99	3, 99	0.5, 101	3, 81 *	4, −174 *
hCB_2_R: beta arrestin EC_50_ (nM), efficacy (%)	101, 107 *	109, 81 *	21, 119 *	55, 51	3, −18
hCB_1_R: beta arrestin EC_50_ (nM)	>10,000 *	>30,000 *	>30,000	>30,000 *	n.d.
hCB_1_R beta arrestin EC_50_/hCB_2_R beta arrestin EC_50_	>99	>275	>1429	>545	n.d.
mouse CB_2_R: beta arrestin EC_50_ (nM), efficacy (%)	592, 76	224, 54	26, 94	>30,000	>30,000

## Supplementary Materials

The following are available online at https://www.mdpi.com/1420-3049/24/18/3338/s1, Figure S1: Overlay of the docking poses from CB_2_R ligands within the binding cavity of the inactive-state CB_2_R X-ray structure, Figure S2: Dose response for the CB_2_R agonist, HU910, Figure S3: Gr-1 antibody neutrophil depletion, Figure S4: Neutrophil depletion and leukocyte adhesion during EIU, Table S1: Physicochemical and early absorption, distribution, metabolism, excretion, and toxicology properties, Table S2: Solubility of CB_2_R ligands in selected solvents, Table S3: Mouse pharmacokinetic profiles of CB_2_R ligands, Table S4: Leukocyte-endothelial rolling during EIU and treatment with CB_2_R ligands.

## References

[1] Guly C.M., Forrester J.V. (2010). Investigation and management of uveitis. BMJ.

[2] Yadav U.C.S., Subramanyam S., Ramana K.V. (2009). Prevention of Endotoxin-Induced Uveitis in Rats by Benfotiamine, a Lipophilic Analogue of Vitamin B1. Invest. Ophthalmol. Vis. Sci..

[3] Lu Y.-C., Yeh W.-C., Ohashi P.S. (2008). LPS/TLR4 signal transduction pathway. Cytokine.

[4] Li S., Lu H., Hu X., Chen W., Xu Y., Wang J. (2010). Expression of TLR4-MyD88 and NF-κB in the iris during endotoxin-induced uveitis. Mediators Inflamm..

[5] Barry R.J., Nguyen Q.D., Wlee R., Imurray P., Denniston A.K. (2014). Pharmacotherapy for uveitis: Current management and emerging therapy. Clin. Ophthalmol..

[6] Kersey J.P., Broadway D.C. (2006). Corticosteroid-induced glaucoma: A review of the literature. Eye.

[7] Pacher P., Batkai S., Kunos G. (2006). The endocannabinoid system as an emerging target of pharmacotherapy. Pharmacol. Rev..

[8] Munoz-Luque J., Ros J., Fernandez-Varo G., Tugues S., Morales-Ruiz M., Alvarez C.E., Friedman S.L., Arroyo V., Jimenez W. (2007). Regression of fibrosis after chronic stimulation of cannabinoid CB2 receptor in cirrhotic rats. J. Pharmacol. Exp. Ther..

[9] Rajesh M., Mukhopadhyay P., Bátkai S., Patel V., Saito K., Matsumoto S., Kashiwaya Y., Horváth B., Mukhopadhyay B., Becker L. (2010). Cannabidiol attenuates cardiac dysfunction, oxidative stress, fibrosis, and inflammatory and cell death signaling pathways in diabetic cardiomyopathy. J. Am. Coll. Cardiol..

[10] Kreitzer F.R., Stella N. (2009). The therapeutic potential of novel cannabinoid receptors. Pharmacol. Ther..

[11] Mechoulam R., Parker L.A. (2013). The endocannabinoid system and the brain. Annu. Rev. Psychol..

[12] McPartland J.M., Duncan M., Di Marzo V., Pertwee R. (2015). Are cannabidiol and Δ(9)-tetrahydrocannabivarin negative modulators of the endocannabinoid system? A systematic review. Br. J. Pharmacol..

[13] Munro S., Thomas K.L., Abu-Shaar M. (1993). Molecular characterization of pheripheral receptor for cannabinoids. Nature.

[14] Galiegue S., Mary S., Marchand J., Dussossoy D., Carriere D., Carayon P., Bouaboula M., Shire D., Le Fur G., Casellas P. (1995). Expression of central and peripheral cannabinoid receptors in human immune tissues and leukocyte subpopulations. Eur. J. Biochem..

[15] Cairns E.A., Toguri T.J., Porter R.F., Szczesniak A.M., Kelly M.E.M. (2016). Seeing over the horizon: Targeting the endocannabiond system for the treatment of ocular disease. J. Basic Clin. Physiol. Pharmacol..

[16] Rom S., Zuluaga-Ramirez V., Dykstra H., Reichenbach N.L., Pacher P., Persidsky Y. (2013). Selective activation of cannabinoid receptor 2 in leukocytes suppresses their engagement of the brain endothelium and protects the blood-brain barrier. Am. J. Pathol..

[17] Tanasescu R., Constantinescu C.S. (2010). Cannabinoids and the immune system: An overview. Immunobiology.

[18] Maresz K., Carrier E.J., Ponomarev E.D., Hillard C.J., Dittel B.N. (2005). Modulation of the cannabinoid CB2 receptor in microglial cells in response to inflammatory stimuli. J. Neurochem..

[19] Storr M.A., Keenan C.M., Zhang H., Patel K.D., Makriyannis A., Sharkey K.A. (2009). Activation of the cannabinoid 2 receptor (CB2) protects against experimental colitis. Inflamm. Bowel Dis..

[20] Horváth B., Magid L., Mukhopadhyay P., Bátkai S., Rajesh M., Park O., Tanchian G., Gao R.Y., Goodfellow C.E., Glass M. (2012). A new cannabinoid CB2 receptor agonist HU-910 attenuates oxidative stress, inflammation and cell death associated with hepatic ischaemia/reperfusion injury. Br. J. Pharmacol..

[21] Di Marzo V. (2008). Targeting the endocannabinoid system: To enhance or reduce?. Nat. Rev. Drug Discov..

[22] Szczesniak A., Porter R., Toguri J., Borowska-Fielding J., Siwakoti A., Lehmann C., Kelly M. (2017). Cannabinoid 2 receptor is a novel anti-inflammatory target in experimental proliferative vitreoretinopathy. Neuropharmcology.

[23] Toguri J.T., Lehmann C., Laprairie R.B., Szczesniak A.M., Zhou J., Denovan-Wright E.M., Kelly M.E.M. (2014). Anti-inflammatory effects of cannabinoid CB2 receptor activation in endotoxin-induced uveitis. Br. J. Pharmacol..

[24] Toguri T., Leishman E., Szczesniak A., Laprairie R., Oehler O., Straiker A., Kelly M.E.M., Bradshaw H.B. (2018). Inflammation and CB2 signaling drive novel changes in the ocular lipidome and regulate immune cell activity in the eye. Prostaglandins Other Lipid Mediat..

[25] Lu H.-C., Mackie K. (2016). An introduction to the endogenous cannabinoid system. Biol. Psychiatry.

[26] Grimaldi P., Di Giacomo D., Geremia R. (2013). The endocannabinoid system and spermatogenesis. Front. Endocrinol. (Lausanne)..

[27] Pertwee R.G. (2012). Targeting the endocannabinoid system with cannabinoid receptor agonists: Pharmacological strategies and therapeutic possibilities. Philos. Trans. R. Soc. B Biol. Sci..

[28] Soethoudt M., Grether U., Fingerle J., Grim T.W., Fezza F., de Petrocellis L., Ullmer C., Rothenhäusler B., Perret C., van Gils N. (2017). Cannabinoid CB2 receptor ligand profiling reveals biased signalling and off-target activity. Nat. Commun..

[29] Bissantz C., Grether U., Kimbara A., Nettekoven M., Roever S., Rogers-Evans M. (2013). Preparation of [1,2,3]triazolo [4,5-d]pyrimidine derivatives useful as cannabinoid receptor 2 agonists.

[30] Grether U., Kimbara A., Nettekoven M., Ricklin F., Roever S., Rogers-Evans M., Rombach D., Schulz-Gasch T., Westphal M. (2014). Pyridine-2-amides useful as CB2 agonists.

[31] Frei B., Gobbi L., Grether U., Ricklin F., Roever S., Rogers-Evans M., Rombach D. (2018). Pyridine derivatives.

[32] Ouali Alami N., Schurr C., Olde Heuvel F., Tang L., Li Q., Tasdogan A., Kimbara A., Nettekoven M., Ottaviani G., Raposo C. (2018). NF-κB activation in astrocytes drives a stage-specific beneficial neuroimmunological response in ALS. EMBO J..

[33] Nettekoven M., Adam J.M., Bendels S., Bissantz C., Fingerle J., Grether U., Grüner S., Guba W., Kimbara A., Ottaviani G. (2016). Novel triazolopyrimidine-derived cannabinoid receptor 2 agonists as potential treatment for inflammatory kidney diseases. Chem. Med. Chem..

[34] Hanus L., Breuer A., Tchilibon S., Shiloah S., Goldenberg D., Horowitz M., Pertwee R.G., Ross R.A., Mechoulam R., Fride E. (1999). HU-308: A specific agonist for CB(2), a peripheral cannabinoid receptor. Proc. Natl. Acad. Sci. USA.

[35] Li X., Hua T., Vemuri K., Ho J.H., Wu Y., Wu L., Popov P., Benchama O., Zvonok N., Locke K. (2019). Crystal Structure of the Human Cannabinoid Receptor CB2. Cell.

[36] Lorenzen E., Sakmar T.P. (2019). Receptor Structures for a Caldron of Cannabinoids. Cell.

[37] Ullmer C., Zoffmann S., Bohrmann B., Matile H., Lindemann L., Flor P.J., Malherbe P. (2012). Functional monoclonal antibody acts as a biased agonist by inducing internalization of metabotropic glutamate receptor 7. Br. J. Pharmacol..

[38] Di L., Rong H., Feng B. (2013). Demystifying brain penetration in central nervous system drug discovery. J. Med. Chem..

[39] Honer M., Gobbi L., Martarello L., Comley R.A. (2014). Radioligand development for molecular imaging of the central nervous system with positron emission tomography. Drug Discov. Today.

[40] Kansy M., Senner F., Gubernator K. (1998). Physicochemical high throughput screening: Parallel artificial membrane permeation assay in the description of passive absorption processes. J. Med. Chem..

[41] De Filippo K., Dudeck A., Hasenberg M., Nye E., van Rooijen N., Hartmann K., Gunzer M., Roers A., Hogg N. (2013). Mast cell and macrophage chemokines CXCL1/CXCL2 control the early stage of neutrophil recruitment during tissue inflammation. Blood.

[42] Becker M.D., Nobiling R., Planck S.R., Rosenbaum J.T. (2000). Digital video-imaging of leukocyte migration in the iris: Intravital microscopy in a physiological model during the onset of endotoxin-induced uveitis. J. Immunol. Methods.

[43] McHugh D., Tanner C., Mechoulam R., Pertwee R.G., Ross R.A. (2008). Inhibition of human neutrophil chemotaxis by endogenous cannabinoids and phytocannabinoids: Evidence for a site distinct from CB1 and CB2. Mol. Pharmacol..

[44] Dubald M., Bourgeois S., Andrieu V., Fessi H. (2018). Ophthalmic drug delivery systems for antibiotherapy- A review. Pharmaceutics.

[45] Benito C., Núñez E., Tolón R.M., Carrier E.J., Rábano A., Hillard C.J., Romero J. (2003). Cannabinoid CB2 receptors and fatty acid amide hydrolase are selectively overexpressed in neuritic plaque-associated glia in Alzheimer’s disease brains. J. Neurosci..

[46] Carrier E.J., Kearn C.S., Barkmeier A.J., Breese N.M., Yang W., Nithipatikom K., Pfister S.L., Campbell W.B., Hillard C.J. (2004). Cultured rat microglial cells synthesize the endocannabinoid 2-arachidonylglycerol, which increases proliferation via a CB2 receptor-dependent mechanism. Mol. Pharmacol..

[47] Eljaschewitsch E., Witting A., Mawrin C., Lee T., Schmidt P.M., Wolf S., Hoertnagl H., Raine C.S., Schneider-Stock R., Nitsch R. (2006). The endocannabinoid anandamide protects neurons during CNS inflammation by induction of MKP-1 in microglial cells. Neuron.

[48] Ramirez B.G. (2005). Prevention of Alzheimer’s disease pathology by cannabinoids: Neuroprotection mediated by blockade of microglial activation. J. Neurosci..

[49] Sardinha J., Kelly M.E.M., Zhou J., Lehmann C. (2014). Experimental cannabinoid 2 receptor-mediated immune modulation in sepsis. Mediators Inflamm..

[50] Lehmann C., Kianian M., Zhou J., Küster I., Kuschnereit R., Whynot S., Hung O., Shukla R., Johnston B., Cerny V. (2012). Cannabinoid receptor 2 activation reduces intestinal leukocyte recruitment and systemic inflammatory mediator release in acute experimental sepsis. Crit. Care.

[51] Xu H., Cheng C.L., Chen M., Manivannan A., Cabay L., Pertwee R.G., Coutts A., Forrester J. (2007). V Anti-inflammatory property of the cannabinoid receptor-2-selective agonist JWH-133 in a rodent model of autoimmune uveoretinitis. J. Leukoc. Biol..

[52] McGrath J.C., Drummond G.B., McLachlan E.M., Kilkenny C., Wainwright C.L. (2010). Editorial: Guidelines for reporting experiments involving animals: The ARRIVE guidelines. Br. J. Pharmacol..

[53] Kilkenny C., Browne W., Cuthill I.C., Emerson M., Altman D.G. (2010). Animal research: Reporting in vivo experiments: The ARRIVE guidelines. Br. J. Pharmacol..

[54] Kezic J., Taylor S., Gupta S., Planck S.R., Rosenzweig H.L., Rosenbaum J.T. (2011). Endotoxin-induced uveitis is primarily dependent on radiation-resistant cells and on MyD88 but not TRIF. J. Leukoc. Biol..

[55] Sun C., Forster C., Nakamura F., Glogauer M. (2013). Filamin-A regulates neutrophil uropod retraction through RhoA during chemotaxis. PLoS ONE.

[56] Tole S., Mukovozov I.M., Huang Y.-W., Magalhaes M.A.O., Yan M., Crow M.R., Liu G.Y., Sun C.X., Durocher Y., Glogauer M. (2009). The axonal repellent, Slit2, inhibits directional migration of circulating neutrophils. J. Leukoc. Biol..

[57] Sieve A.N., Meeks K.D., Bodhankar S., Lee S., Kolls J.K., Simecka J.W., Berg R.E. (2009). A novel IL-17-dependent mechanism of cross protection: Respiratory infection with mycoplasma protects against a secondary listeria infection. Eur. J. Immunol..

[58] Daley J.M., Thomay A.A., Connolly M.D., Reichner J.S., Albina J.E. (2007). Use of Ly6G-specific monoclonal antibody to deplete neutrophils in mice. J. Leukoc. Biol..

