# Supplementary material for: Selective Cannabinoid 2 Receptor Agonists as Potential Therapeutic Drugs for the Treatment of Endotoxin-Induced Uveitis
Source: Molecules. 2019 Sep 13;24(18):3338. doi: 10.3390/molecules24183338 (PMC6767236; doi:10.3390/molecules24183338)
Supplement: Supplementary file 1 [file molecules-24-03338-s001.pdf]

Supplemental Material

Supplemental Methods:

Molecular Docking

The X-ray structure of CB_2R complexed with the antagonist AM10257 (5zty) was used as a template to dock CB_2R ligands. The docking experiments were performed with the software GOLD (Chemical Computing Group) with default settings [1]. The best 10 docking poses for each compound were energy-minimized within the binding pocket using MOE (molecular operating environment ver. 2014.09, Chemical Computing Group, Montreal, Canada) and examined visually to select the most reasonable docking mode with respect to molecular interactions and internal conformational strain. The final selection was based on checking consistency with the available structure–activity relationship information.

Kinetic Lyophilization Solubility Assay

The solubility of a test compound in phosphate buffer at pH 6.5 from evaporated DMSO stock solution is measured over time, resulting in the kinetic solubility of the compound. Samples were prepared in duplicate from 10 mM DMSO stock solutions. DMSO was evaporated (1 h) with a centrifugal vacuum evaporator (Genevac Technologies). The residue was dissolved in 0.05 M phosphate buffer (pH 6.5), stirred for 1 h, and shaken for 2 h. Twelve hours later the solutions were filtered using a microtiter filter plate (Millipore MSDV N65). The filtrate and its 1:10 dilution were analyzed by direct UV measurement or by HPLC-UV. A four-point calibration curve was prepared from the 10 mM DMSO stock solutions and used to determine the solubility of the compounds. Starting from 10 mM stock solution, the measurement range for molecular weight 500 was 0 to 666 µg/mL.

Saturation Solubility (= Thermodynamic Solubility [2])

Approximately 8.6 mg test compound per milliliter solvent/vehicle was stirred in HPLC vials (9 × 12 × 32 mm, Waters) at 350 rpm for 15 h. Samples of 10 µL each were taken and microscopically examined for presence of solid particles. If the active pharmacological ingredient (API) dissolved completely, more solid API was added, and stirring was continued for another 15 h. Addition of solid material was repeated up to 96 h or until residual solid particles could be detected. Samples of 0.5 mL were then transferred to Eppendorf Ultrafree filter tubes (Filter: PVDF 0.22 µm) and centrifuged at 14,500 rpm for 10 min. The filtrates were diluted in ethanol and analyzed for content by UPLC. The measurements were also conducted in 0.05 M aqueous phosphate buffer, fasted (FaSSIF), and fed (FeSSIF) simulated gastrointestinal fluid [3].

Chemical Stability (= Aqueous Stability Assay ASTA)

ASTA is a fully automated assay to determine the 2 h stability of a molecule in aqueous conditions, over the pH range 1–10. Five different pH values are used and the solutions are heated at 37 °C. A compound is classified unstable if, after 2 h, less than 90% of the initial concentration can be found. Samples are first prepared on incubation plates and shaken initially for 10 min at 37 °C. Solutions are then transferred to a filter plate (Millipore MSGVN2250, pore size 0.22 µm) and filtrated into V-bottom plates (from ABGene, AB-0800) that are heat-sealed prior to HPLC analysis. The procedure is performed a second time. However, the incubation time at 37 °C for the second plate is increased by 2 h. Samples are taken at time point 0 and 2 h and analyzed by HPLC.

Passive Membrane Permeability

The parallel artificial membrane permeability assay is a method which determines the permeability of substances from a donor compartment, through a lipid-infused artificial membrane into an acceptor compartment [4]. Read-out is a permeation coefficient P_{eff} drug as well as test compound concentrations in donor, membrane, and acceptor compartments.

A 96-well microtiter plate completely filled with aqueous buffer solutions (pH 7.4/6.5) is covered with a microtiter filter plate in a sandwich construction. The hydrophobic filter material (Durapore/Millipore; pore size 0.22–0.45 μm) of the first 48 wells (sample) of the filter plate is impregnated with 1%–20% solution of lecithin in an organic solvent (dodecane, hexadecane, 1,9-decadiene). The filter surface of the remaining 48 wells (reference) is wetted with a small volume (4–5 μL) of a 50% (*v/v*) methanol/buffer solution. Transport studies were started by the transfer of 100–200 μL of a 250 or 500 μM stock solution on top of the filter plate in the sample and in the reference section, respectively. In general, 0.05 M TRIS, pH 7.4, or 0.05 M phosphate, pH 6.5, buffers were used. The maximum DMSO content of the stock solutions was 5%.

Microsomal Clearance

For human and mice, pooled commercially-available microsome preparations from liver tissues were used (BD UltraPool HLM 150) [5]. For human, ultra-pooled (150 mixed gender donors) liver microsomes were purchased to account for the biological variance in vivo. For the microsome incubations, 96-deep-well plates were applied, which are incubated at 37 °C on a TECAN liquid handling system (Tecan Group Ltd., Switzerland) equipped with Te-Shake shakers and a warming device (Tecan Group Ltd., Switzerland). The incubation buffer was 0.1 M phosphate buffer at pH 7.4. The NADPH regenerating system consisted of 30 mM glucose-6-phosphate disodium salt hydrate; 10 mM NADP; 30 mM $MgCl_2 \times 6 H_2O$; and 5 mg/mL glucose-6-phosphate dehydrogenase (Roche Diagnostics) in 0.1 M potassium phosphate buffer pH 7.4.

Incubations of a test compound at 1 μM in microsome incubations of 0.5 mg/mL plus cofactor NADPH were performed in 96-well plates at 37 °C. After 1, 3, 6, 9, 15, 25, 35, and 45 min, 40 μL incubation solutions are transferred and quenched with 3:1 (*v/v*) acetonitrile containing internal standards. Samples were then cooled and centrifuged before analysis by LC-MS/MS. Log peak area ratios (test compound peak area/internal standard peak area) were plotted against incubation time using a linear fit. The calculated slope was used to determine the intrinsic clearance: Clint (μL/min/mg protein) = –slope (min–1) × 1000/[protein concentration (mg/mL)].

Hepatocyte Clearance

For animals, hepatocyte suspension cultures were either freshly prepared by liver perfusion or prepared from cryopreserved hepatocyte batches. For human, commercially-available, pooled (5–20 donors), cryopreserved human hepatocytes from non-transplantable liver tissues were used [6]. For suspension cultures, Nunc U96 PP-0.5 mL (Nunc Natural, 267245) plates were incubated in a Thermo Forma incubator from Fischer Scientific (Wohlen, Switzerland) equipped with shakers from Variomag® Teleshake shakers (Sterico, Wangen, Switzerland) for maintaining cell dispersion. The cell culture medium was William's media supplemented with Glutamine, antibiotics, insulin, dexamethasone, and 10% FCS.

Test compounds were incubated at a concentration of 1 μM in 96-well plates containing suspension cultures 1×10^6 hepatocytes/mL (~1 mg/mL protein concentration) Plates were shaken at 900 rpm for up to 2 h in a 5% CO_2 atmosphere and 37 °C. After 3, 6, 10, 20, 40, 60, and 120 min, the 100 μL cell suspension in each well was quenched with 200 μL methanol containing an internal standard. Samples were then cooled and centrifuged before analysis by LC-MS/MS.

Log peak area ratios (test compound peak area/internal standard peak area) or concentrations were plotted against incubation time and a linear fit made to the data with emphasis upon the initial rate of compound disappearance. The slope of the fit was then used to calculate the intrinsic clearance: Clint (μL/min/1 × 106 cells) = –slope (min–1) × 1000/[1 × 106 cells].

Plasma Protein Binding

Pooled and frozen plasma from selected species were obtained from commercial suppliers (human HMPLEDTA and mouse MSEPLEDTA3-C57; BioreclamationIV, NY, USA) [7,8]. Teflon equilibrium dialysis plates (96-well, 150 µL, half-cell capacity) and cellulose membranes (12–14 kDa molecular weight cut-off) were purchased from HT-Dialysis (Gales Ferry, Connecticut). Both biological matrix and phosphate buffer pH were adjusted to 7.4 on the day of the experiment. The reference substance was diazepam.

The determination of unbound compound was performed using a 96-well format equilibrium dialysis device with a molecular weight cut-off membrane of 12 to 14 kDa. The Teflon equilibrium dialysis device minimizes non-specific binding of the test substance. Compounds were tested in cassettes of 2 to 5 with an initial total concentration of 1000 nM, one of the cassette compounds being the positive control diazepam. Equal volumes of matrix samples containing substances and blank dialysis buffer (Soerensen buffer at pH 7.4) were loaded into the opposite compartments of each well. The dialysis block was sealed and incubated for 5 h at a temperature of 37 °C and 5% CO_2. After this time, equilibrium will have been reached for the majority of small molecule compounds with a molecular weight of <600. The seal was then removed and matrix and buffer from each dialysis was prepared for analysis by LC-MS/MS. All protein binding determinations were performed in triplicate. The integrity of membranes was tested in the HTDialysis device by determining the unbound fraction values for the positive control diazepam in each well.

At equilibrium, the unbound drug concentration in the biological matrix compartment of the equilibrium dialysis apparatus is the same as the concentration of the compound in the buffer compartment. Thus, the percent unbound fraction was be calculated by determining the compound concentrations in the buffer and matrix compartments after dialysis as follows: % fraction unbound = 100 × buffer concentration after dialysis/matrix concentration after dialysis. The device recovery was checked by measuring the compound concentrations in the matrix before dialysis and calculating the percent recovery (mass balance). Recovery had to be within 80% to 120% for data acceptance.

P-Glycoprotein (P-gp) Assay

P-glycoprotein (permeability-glycoprotein (P-gp), also known as multidrug resistance protein 1) is the most studied and best characterized drug transporter. The P-gp assay evaluates the ability of test compounds to serve as a P-gp substrate [9]. The assay uses transfected LLC-PK1 cells (porcine kidney epithelial cells) expressing human or mouse P-gp, cultured on 96-well semi-permeable filter membrane plates. Cells form a polarized monolayer with tight junctions, and act as a barrier between apical and basolateral compartments. P-gp is expressed in the apical-facing membrane of the monolayer (tightness confirmed using Lucifer yellow). For substrate testing, the assay determines the unidirectional permeability (Papp) of a test compound by separately dosing the apical (for A > B Papp) and basolateral (for B > A Papp) sides of the cell monolayer (i.e., donor compartments), and measuring the movement of the compound into the respective receiver compartments over a 3 h incubation at 37 °C. The effect of P-gp was measured by expressing the efflux ratio of the unidirectional A > B and B > A Papp values. The mean permeability (A > B and B > A Papp) was determined in the absence of P-gp via addition of the selective inhibitor zosuquidar. The efflux ratio and mean Papp were then used to categorize compounds based on their degree of efflux and passive permeability.

Cytochrome P450 (CYP) 3A4, 2C9, and 2D6 Inhibition Assay

The experiments aim to allow some estimation of drug–drug interaction risk, where a compound inhibits one or more cytochrome P450 (CYP) enzymes that are responsible for the metabolism of a co-medicated drug molecule. The assays typically generate two endpoints: IC_{50} value (µM) and percent inhibition at highest acceptable test concentration (typically 50µM, lower if highest concentration data rejected due to insolubility). Experimental details of the MS-based method have been described in Fowler and Zhang et al. 2008 [10].

Glutathione (GSH) Adduct Formation in Human Liver Microsomes

The GSH adduct formation test aims for the identification of molecules which bear the risk of forming reactive metabolites that might trigger drug-induced liver injury and drug-induced hypersensitivity reactions in patients. The compounds are incubated with liver microsomes to assess reactive metabolite formation. Glutathione is added as a nucleophile to convert eventually formed reactive species into a stable conjugate that can be analyzed by mass spectrometry. Details on the assay have been reported in Brink et al. (2014) [11].

Mouse Pharmacokinetic Profiles

Male C57Bl/6 mice (Charles River, France) were used to study the pharmacokinetics of CB_2R ligands after i.v. and p.o. administration. Animals were up to 12 weeks of age. All animal studies in this section were performed at Hoffmann-La Roche and approved by the Federal Food Safety and Veterinary Office of the Swiss Confederation. Test compounds were formulated according to respective protocols either by dissolution (i.v.) or as aqueous suspensions in a glass potter until homogeneity was achieved (p.o.; formulated as a solution in ethanol/cremophor EL/ 0.9% NaCl (5%/5%/90%)). Formulations were injected i.v. using a 30G needle in the lateral tail vein of mice using a volume of 50 μL in the dose indicated. For p.o. applications, animals were gavaged using a volume of 100 μL in the dose indicated. At the following time points blood was drawn into EDTA: 0.08, 0.25, 0.5, 1, 2, 4, 7 h (for p.o. the first time point was omitted). Six animals were used for each compound. Animals were distributed randomly over the time course and at each time point, a volume of 100 μL of blood was taken. Quantitative plasma measurement of the compound was performed by LC-MS/MS analysis. Pharmacokinetic analysis was performed using Phoenix WinNonlin 6.4 software using a non-compartmental approach consistent with the route of administration. For assessment of the exposure, Cmax, Tmax, and area under the curve (AUC) were determined from the serum concentration profiles. Parameters (clearance, versus, T1/2) were estimated using nominal sampling times relative to the start of each administration. C_0 (initial concentration) was extrapolated from the first concentration measured following i.v. administration.

Supplemental Results:

Solubility

The CB_2R ligands examined exhibited different solubility characteristics in different solvent systems (Supplemental Table 2). Tocrisolve™ 100 (Tocris Bioscience) proved to be a good solvent for HU910, RO6851228, and RO6871085. The solubility of RO6871304 and HU308 were considerably lower in this solvent. Dissolution kinetics of HU910 in Tocrisolve™ 100 was comparatively slow. Full dissolution could only be achieved after approximately 72 h of stirring (data not shown).

In addition to topical administration, these drugs also have favorable PK for other routes of administration (Supplemental Table 3). After i.v. (0.5–1 mg/kg) or peroral (3 mg/kg) administration in mice, the cannabinoid ligands tested reach peak concentration approximately 0.25 to 1 h after administration, have low clearance rate, good volume of distribution (Vss; 0.95–4 (L/kg)), and good half-lives (0.3 to 4.15 h). Since the route of administration used for ocular delivery in this study was topical the PK data values are assumed to be lower but still have good bioavailability. Furthermore, we assume these ligands reach high concentration within the anterior ocular tissues.

Supplemental Figures and Tables:

A: Overlay of the docking poses from RO6871304 (bronze), RO6871085 (green), and HU910 (red) within the binding cavity of the inactive-state CB$_2$R X-ray structure.

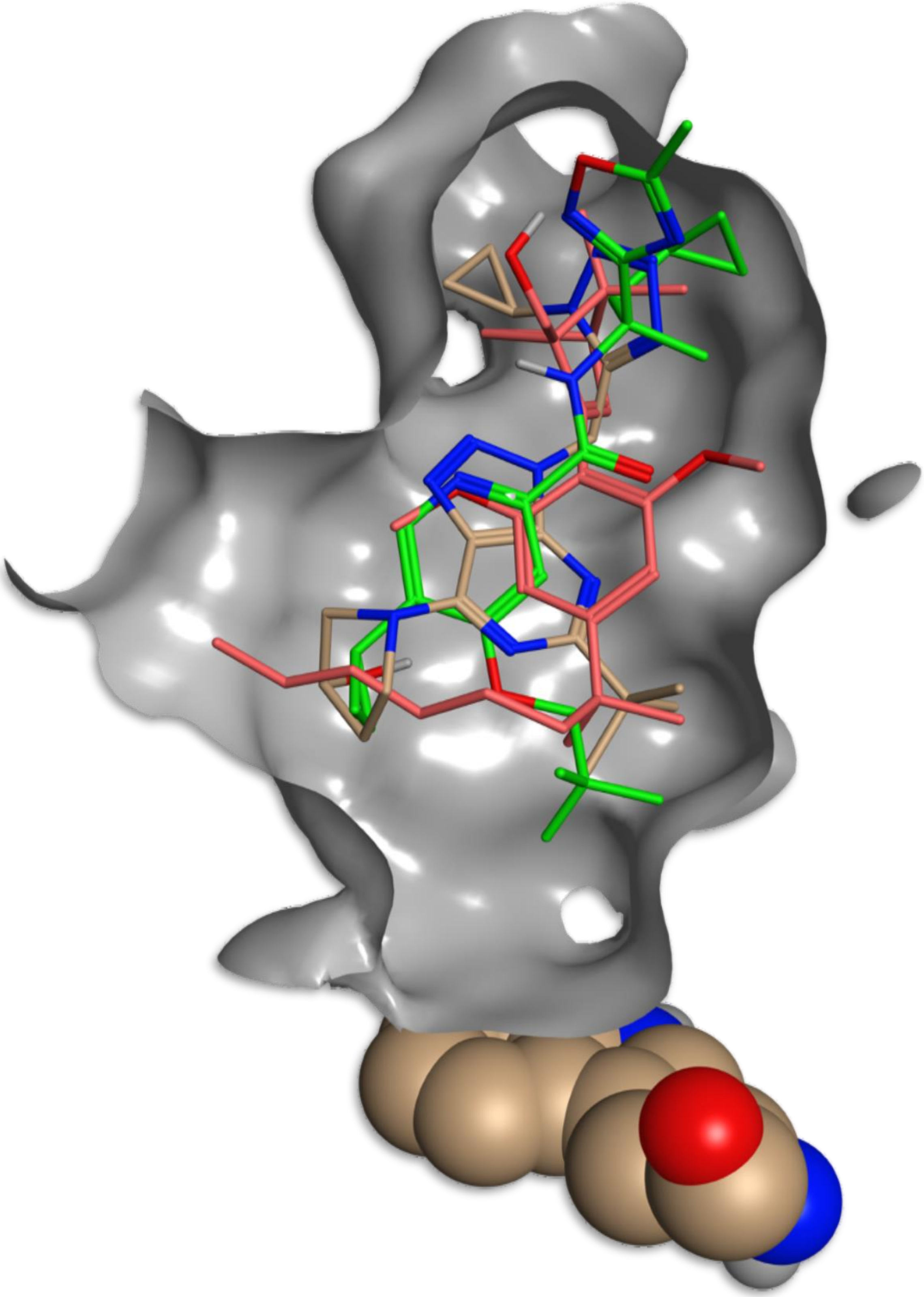

B: CB$_2$R inverse agonist RO6851228 docked into the binding cavity of the inactive-state CB$_2$R X-ray structure.

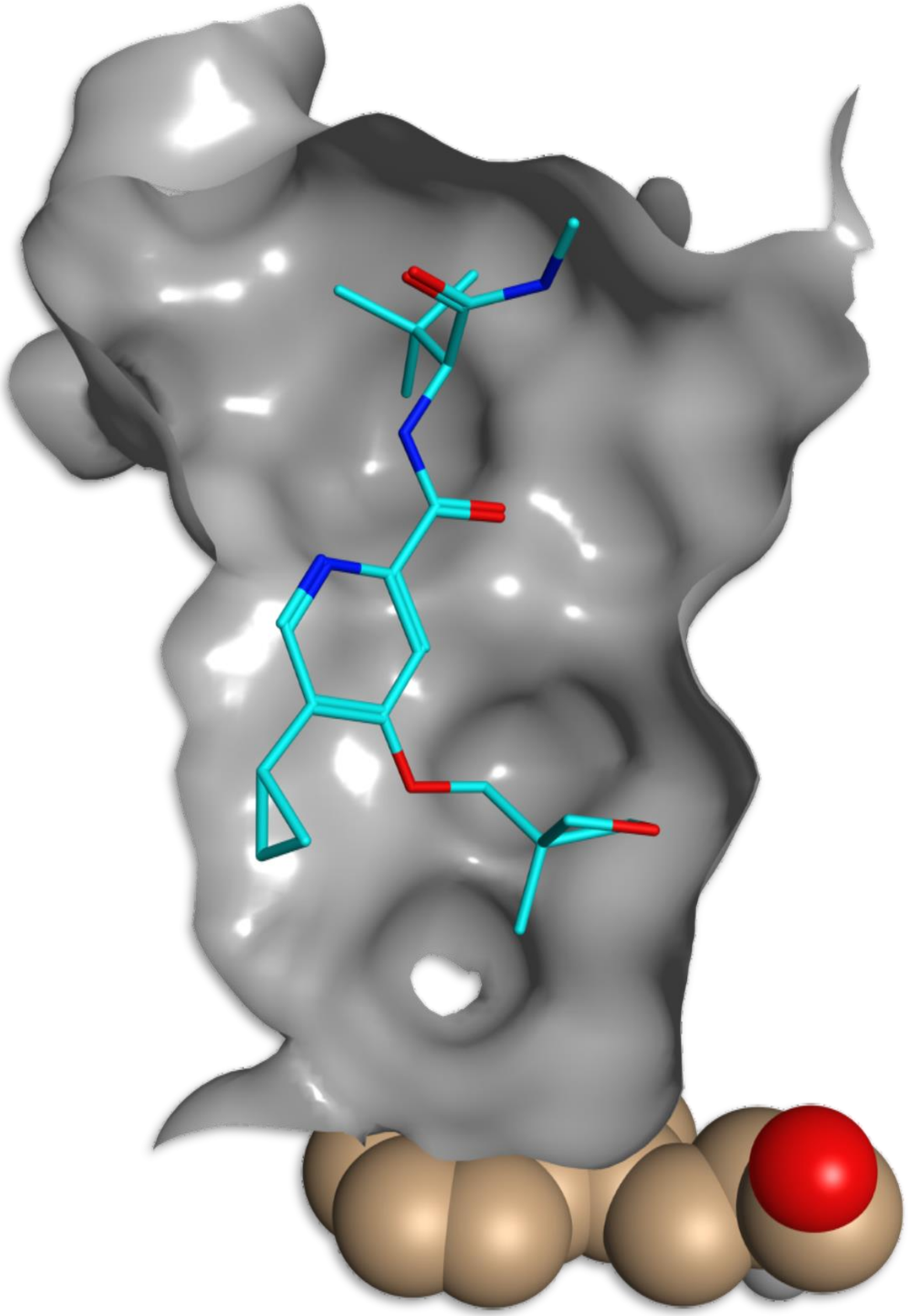

Supplemental Figure 1: Overlay of the docking poses from triazolopyrimidine RO6871304 (bronze), 2,4,5-trisubstituted pyridine RO6871085 (green), and HU910 (red) within the binding cavity of the inactive-state CB_2R X-ray structure [12] **(A).** Docking pose of CB_2R inverse agonist RO6851228 in the binding cavity of the inactive-state CB_2R X-ray structure [12] (**B**). At the bottom of the graphs toggle switch residue Trp258$^{6.48}$ is depicted (bronze) [12].

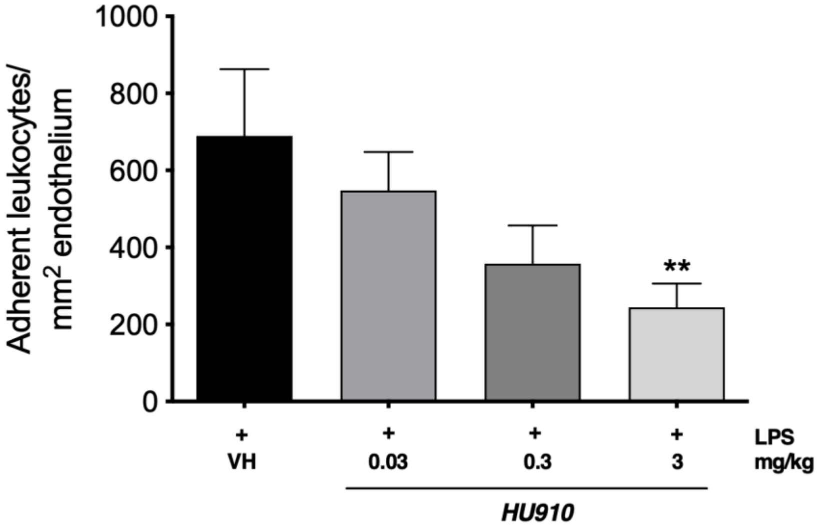

Supplemental Figure 2: Dose response for the CB_2R agonist, HU910. Bar graph represents the mean number of adherent leukocytes 6 h after intravitreal injection of: LPS (250 ng) + vehicle (i.v.; micelles; n = 6) or LPS + HU910 (i.v.; 0.03–3.0 mg/kg; n = 6). Data are presented as mean ± SD. One-way ANOVA with Dunnett; ** $p < 0.01$ compared to LPS + vehicle.

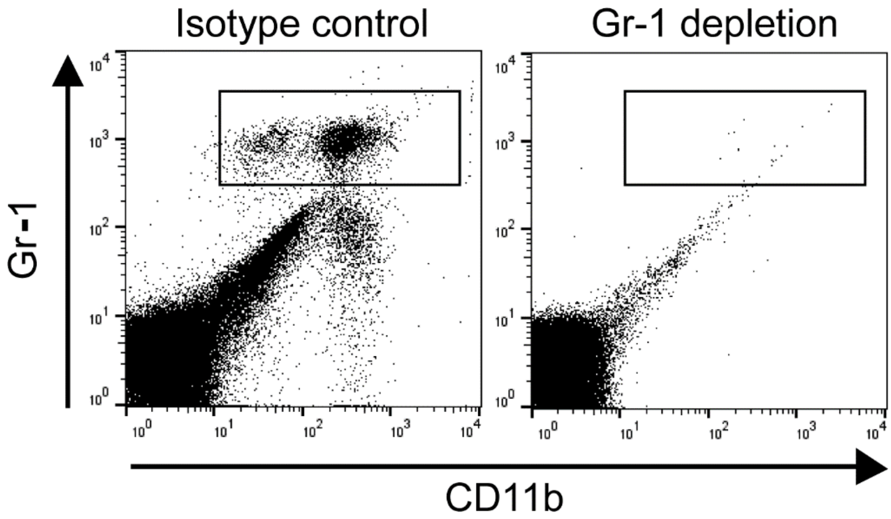

Supplemental Figure 3: Gr-1 antibody depletes neutrophils in the peripheral blood after 24 h. Flow cytometry analysis of peripheral blood for Gr-1^{+} cells from mice following in vivo administration of neutrophil-depleting Gr-1 antibody. WT mice injected with isotype control or 50 µL Gr-1 i.p. 24 h before FACS analysis and generation of EIU. Representative example. n = 2.

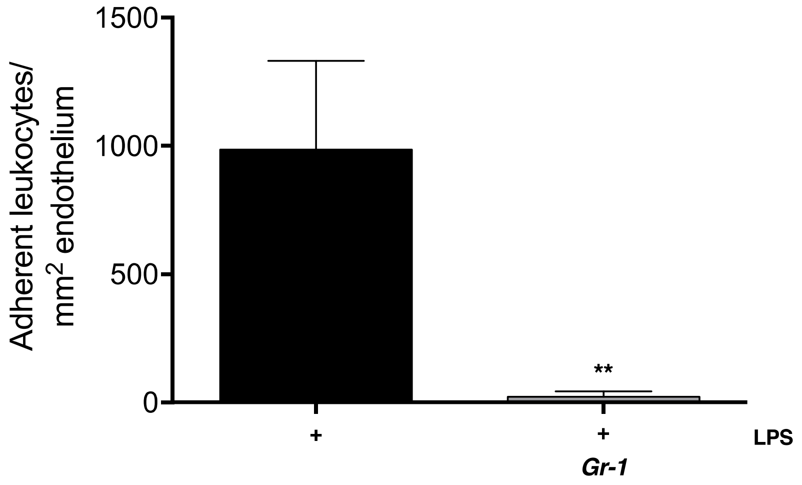

Supplemental Figure 4: Neutrophil depletion abolishes leukocyte adhesion to iridial microvasculature at 6 h post-EIU induction. Bar graph represents mean number of adherent leukocytes in the iris microcirculation of isotype control and Gr-1 antibody-treated mice at 6 h post-LPS injection. Rhodamine 6G was used to fluorescently label autologous leukocytes 15 min prior to IVM analysis. n = 6–8, two-tailed unpaired t-test; ** $p < 0.01$ compared to LPS + isotype control.

Supplemental Table 1: Calculated physicochemical and early absorption, distribution, metabolism, excretion, and toxicology properties with relevance for good in vivo performance of CB_2R agonists, HU308, HU910, RO6871304, RO6871085, and CB_2R inverse agonist RO6851228. (Partially published for HU308 and HU910 in [13], for RO6871304 in [14], and for RO6871085 as well as RO6851228 in [15]). Due to their relevance for the described pharmacology studies they are provided here in a condensed format. * Indicates previously-published data.

Compound	HU308	HU910	RO6871304	RO6871085	RO6851228
**Molecular weight (MW) (g/mol)**	414.6*	414.6*	384.5*	424.4*	389.5
**TPSA (Å^2)**	27.5*	31.4*	115.9	72.3	74.8
**Number of Hydrogen-Bond Donors (HBD)**	1	1	1*	1*	2*
**Kow clog*P***	8.97*	9.00*	0.99*	5.13	2.82
**MP (°C)**	n.d.	59.4	142.8	n.d.	n.d.
**HSA (A^2)**	386.7	389.9	247.8	319.0	308.6
**Log*D* (at pH 7.4)**	4.29	out of range	2.78*	3.94*	3.21*
**pK_a**	n.d.	n.d.	2.7 (basic)	2.6 (basic, calculated), 10.1 (acidic, calculated)*	3.3 (basic)*
**Kinetic Solubility (µg/mL)**	<0.75*	<0.5*	162 ± 2*	4.2 ± 0.1	174 ± 47
**Thermodynamic Solubility in Aqueous Buffer (pH 6.5)/FaSSIF (pH 6.5)/FeSSIF (pH 5.5) (µg/mL)**	<1/52/60	<1/59/128	56/80/273*	3/37/56	497/421/243
**Chemical Stability in Aqueous Buffer at pH 1/4/6.5/8/10 (% remaining after 2 h at 37 °C)**	n.d.	94/93/91/94/93	100/100/100/99/100	99/99/97/99/99	93/100/99/100/100
**PAMPA P_{eff} (10*-6 cm/s), %Acceptor/%Membrane/%Donor**	2.53 3/74/23*	0.45 1/57/42*	3.06 8/30/62*	2.44 2/78/20*	5.17 19/3/79*
**Microsomal Clearance human/mouse (µL/min/kg)**	n.d./n.d.*	90/464*	12/71*	<10/26*	34/53*
**Hepatocyte Clearance human/mouse (µL/min/Mio cells)**	8.3/4.7*	9.2/34*	15/140*	17/27*	12/46*
**Plasma Protein Binding: FREE Fraction (%) human/mouse**	n.d./n.d*.	1.8/4.1*	13/3.1	1.0/0.9*	9.6/n.d.*
**P-glycoprotein-Mediated Efflux Extraction Ratio human/mouse**	6.2/7.3*	2.2/3.0*	n.d./n.d.	1.4/1.7*	6.5/25.1*

**IC50 CYP3A4/CYP2C9/CYP 2D6 (μM)**	>50/>50/>50	37/>50/>50	>50/16/>50	37/8/34	>50/>50/>50
**GSH Adduct Formation in Human Liver Microsomes**	no adduct	no adduct	no adduct	no adduct	no adduct

Supplementary Table 2: Solubility of CB_2R ligands in selected solvents (n = 1). [1] Hydrochloride salt. [2] Crystalline material. [3] In demin. water. [4] > indicates that saturation solubility could not be achieved due to limited amount of available compound. [5] 200 mM glycocholic acid, 200 mM lecithin in water for injection, pH 6. [6] Not used in this study.

	**HU910**		**RO6851228**		**RO6871085**[1]		**RO6871304**		**HU308**[2]	
**Solvent**	**[mg/mL]**	**pH**	**[mg/mL]**	**pH**	**[mg/mL]**	**pH**	**[mg/mL]**	**pH**	**[mg/mL]**	**pH**
Tocrisolve™	19.2	6.2	15.0	7.3	>30.0[4]	1.2	1.3	5.7	1.6	6.1
PEG400 30%[6,3]	< LOD	6.0	5.9	7.4	n.d.	n.d.	0.2	6.1	0.0	5.1
HP-beta-CD 15%[6,3]	0.32	6.2	35.5	7.6	n.d.	n.d.	7.2	6.0	0.02	5.7
MCT (Miglyol 812)[6]	>87.8[4]	n.a.	>19.0[4]	n.a	2.3	n.a	2.3	n.a	>10.3[4]	n.a
Mixed Micelles[5]	4.4	6.2	>1.6	6.6	0.2	6.1	>1.8	6.6	n.d.	n.d.

Supplemental Table 3: Mouse pharmacokinetic profiles of CB_2R ligands HU308, HU910, RO6871304, RO6871085, and RO6851228 following peroral and intravenous administration. n = 6 (in parts published for HU308 and HU910 in [13], for RO6871304 in [14], and for RO6871085 as well as RO6851228 in [15]). Due to their relevance for the described pharmacology studies they are provided here in a condensed format. * Indicates previously published data.

Compound	HU308	HU910	RO6871304	RO6871085	RO6851228
Intravenous Dose (mg/kg)	2	1*	0.5*	0.5*	1*
Clearance (CL (mL/min/kg)	36.6	2.8*	36.5*	11.8*	73.8*
$T_{1/2}$ (h)	2.15	7.4*	0.32	4.15*	1.08*
Vss (L/kg)	2.9	0.26*	0.95*	4.0*	3.1*
AUC_{INF} D (ng*h*kg/mL/mg)	456	6060*	456	1412*	226*
Oral Dose (mg/kg)	5*	3*	n.d.	3*	3*
C_{max} D (ng/mL)	201*	495*	n.d.	176.8*	97.5*
T_{max} (h)	0.5*	1*	n.d.	0.5*	0.25*
$T_{1/2}$ (h)	3.3*	5.2*	n.d.	8.42*	1.17*
AUC_{INF} D (ng*h/mL)	298*	904*	n.d.	729*	70*
F (%)	n.d.	n.d.	n.d.	68*	31*

Supplemental Table 4: Leukocyte-endothelial rolling in the iridial microcirculation 6 h after intravitreal injection of lipopolysaccharide (LPS) and treatment with a CB_2R agonist (RO6871304) or inverse agonist (RO6851228). Figure shows the mean number of rolling leukocytes per min after intravitreal injection of: Saline + vehicle (Topical; n = 6), LPS + vehicle (n = 6), LPS + RO6871304 (1.5% *w*/*v*; n = 6), or LPS + RO6851228 (1.5% *w*/*v*; n = 6). Data are represented as mean ± SD. No significant differences between groups were found. One-way ANOVA with Dunnet; $p > 0.05$ compared to LPS + vehicle.

	**Saline**	**LPS**	**LPS + RO6871304**	**LPS + RO6851228**
**Rolling leukocytes per min**	0.15 ± 0.27	0.38 ± 0.59	0.05 ± 0.07	0.45 ± 0.47

References:

1. Jones, G.; Willett, P.; Glen, R.C.; Leach, A.R.; Taylor, R. Development and validation of a genetic algorithm for flexible docking. *J. Mol. Biol.* **1997**, *267*, 727–748.
2. Kerns, E.H.; Di, L.; Carter, G.T. In vitro solubility assays in drug discovery. *Curr. Drug Metab.* **2008**, *9*.
3. Jakubiak, P.; Wagner, B.; Grimm, H.P.; Petrig-Schaffland, J.; Schuler, F.; Alvarez-Sánchez, R. Development of a unified dissolution and precipitation model and its use for the prediction of oral drug absorption. *Mol. Pharm.* **2016**, *13*, 586–598.
4. Kansy, M.; Senner, F.; Gubernator, K. Physicochemical high throughput screening: Parallel artificial membrane permeation assay in the description of passive absorption processes. *J. Med. Chem.* **1998**, *41*, 1007–1010.
5. Di, L.; Keefer, C.; Scott, D.O.; Strelevitz, T.J.; Chang, G.; Bi, Y.A.; Lai, Y.; Duckworth, J.; Fenner, K.; Troutman, M.D.; et al. Mechanistic insights from comparing intrinsic clearance values between human liver microsomes and hepatocytes to guide drug design. *Eur. J. Med. Chem.* **2012**, *57*, 441–448.
6. LeCluyse, E.L.; Witek, R.P.; Andersen, M.E.; Powers, M.J. Organotypic liver culture models: Meeting current challenges in toxicity testing. *Crit. Rev. Toxicol.* **2012**, *42*, 501–548.
7. Banker, M.J.; Clark, T.H.; Williams, J.A. Development and validation of a 96-well equilibrium dialysis apparatus for measuring plasma protein binding. *J. Pharm. Sci.* **2003**, *92*, 967–974.
8. Zamek-Gliszczynski, M.J.; Ruterbories, K.J.; Ajamie, R.T.; Wickremsinhe, E.R.; Pothuri, L.; Rao, M.V.S.; Basavanakatti, V.N.; Pinjari, J.; Ramanathan, V.K.; Chaudhary, A.K. Validation of 96-well Equilibrium Dialysis with Non-radiolabeled Drug for Definitive Measurement of Protein Binding and Application to Clinical Development of Highly-Bound Drugs. *J. Pharm. Sci.* **2011**, *100*, 2498–2507.
9. Poirier, A.; Cascais, A.C.; Bader, U.; Portmann, R.; Brun, M.E.; Walter, I.; Hillebrecht, A.; Ullah, M.; Funk, C. Calibration of in vitro multidrug resistance protein 1 substrate and inhibition assays as a basis to support the prediction of clinically relevant interactions in vivo. *Drug Metab. Dispos.* **2014**, *42*, 1411–1422.
10. Fowler, S.; Zhang, H. In vitro evaluation of reversible and irreversible cytochrome P450 inhibition: Current status on methodologies and their utility for predicting drug–drug interactions. *AAPS J.* **2008**, *10*, 410–424.
11. Brink, A.; Fontaine, F.; Marschmann, M.; Steinhuber, B.; Cece, E.N.; Zamora, I.; Pähler, A. Post-acquisition analysis of untargeted accurate mass quadrupole time-of-flight MSE data for multiple collision-induced neutral losses and fragment ions of glutathione conjugates. *Rapid Commun. Mass Spectrom.* **2014**, *28*, 2695–2703.
12. Li, X.; Hua, T.; Vemuri, K.; Ho, J.H.; Wu, Y.; Wu, L.; Popov, P.; Benchama, O.; Zvonok, N.; Locke, K.; et al. Crystal Structure of the Human Cannabinoid Receptor CB2. *Cell* **2019**, *176*, 459–467.e13.
13. Soethoudt, M.; Grether, U.; Fingerle, J.; Grim, T.W.; Fezza, F.; de Petrocellis, L.; Ullmer, C.; Rothenhäusler, B.; Perret, C.; van Gils, N.; et al. Cannabinoid CB2 receptor ligand profiling reveals biased signalling and off-target activity. *Nat. Commun.* **2017**, *8*, 1–14.
14. Bissantz, C.; Grether, U.; Kimbara, A.; Nettekoven, M.; Roever, S.; Rogers-Evans, M. Preparation of [1,2,3]triazolo [4,5-d]pyrimidine derivatives useful as cannabinoid receptor 2 agonists. WO 2013076182 A1. 30 May 2013.

15. Ouali Alami, N.; Schurr, C.; Olde Heuvel, F.; Tang, L.; Li, Q.; Tasdogan, A.; Kimbara, A.; Nettekoven, M.; Ottaviani, G.; Raposo, C.; et al. NF-κB activation in astrocytes drives a stage-specific beneficial neuroimmunological response in ALS. *EMBO J.* **2018**, e98697.
